# Potential establishment and ecological effects of bighead and silver carp in a productive embayment of the Laurentian Great Lakes

**DOI:** 10.1007/s10530-020-02263-z

**Published:** 2020-04-21

**Authors:** Lori N. Ivan, Doran M. Mason, Hongyan Zhang, Edward S. Rutherford, Tim Hunter, Shaye Sable, Aaron T. Adamack, Kenneth Rose

**Affiliations:** 1 Quantitative Fisheries Center, Michigan State University, 375 Wilson Road, 101 UPLA Building, East Lansing, MI 48824-1101 USA; 2 NOAA Great Lakes Environmental Research Laboratory, 4840 S. State Rd, Ann Arbor, MI 48108 USA; 3 Eureka Aquatic Research, LLC, Ann Arbor, MI 48108 USA; 4 Dynamic Solutions, LLC, 450 Laurel St, Ste. 1650, Baton Rouge, LA 70801 USA; 5 Fisheries and Oceans Canada, Northwest Atlantic Fisheries Centre, 80 E. White Hills Rd, St. John’s, NL A1A 5J7 Canada; 6 Horn Point Laboratory, University of Maryland Center for Environmental Science, P.O. Box 775, Cambridge, MD 21613 USA

**Keywords:** Ecological modeling, Individual-based modeling, Invasive species, Bigheaded carp, Saginaw Bay, Lake Huron

## Abstract

Bighead carp *H*. *nobilis* and silver carp *Hypothalmichthys molitrix* (collectively bigheaded carps, BHC) are invasive planktivorous fishes that threaten to enter the Laurentian Great Lakes and disrupt food webs. To assess the likelihood of BHC establishment and their likely effects on the food web of Saginaw Bay, Lake Huron, we developed a multi-species individual-based bioenergetics model that tracks individual bighead and silver carp, four key fish species, and seven prey biomass groups over 50 years. The model tracks the daily consumption, mortality and growth of all individuals and the biomass dynamics of interacting prey pools. We ran simulation scenarios to determine the likelihood of BHC establishment under initial introductions from 5 to 1 million yearling and older individuals, and assuming variable age-0 carp survival rates (high, intermediate, and low). We bounded the survival of age-0 BHC as recruitment continues to be one of the biggest unknowns. We also simulated the potential effects of an established population of 1 million bighead carp or silver carp assuming variation in age-0 survival. Results indicated that as few as 10 BHC could establish a population assuming high or intermediate age-0 survival, but at least 100,000 individuals were needed to establish a population assuming low age-0 survival. BHC had negative effects on plankton and planktivorous fish biomass, which increased with BHC density. However, piscivorous walleye *Sander vitreus* appeared to benefit from BHC establishment. The potential for BHC to establish and affect ecologically and economically important fish species in Saginaw Bay is a cause for concern.

**Electronic supplementary material:**

The online version of this article (10.1007/s10530-020-02263-z) contains supplementary material, which is available to authorized users.

## Introduction

Over 180 non-indigenous aquatic species, including viruses, emergent and submergent plants, phytoplankton, zooplankton, benthic macroinvertebrates and fishes, have become established in the Laurentian Great Lakes (Pagnucco et al. 2015; Ricciardi 2006; Sturtevant et al. 2014). Although the majority of these species have remained at very low abundance and/or had little or no detected effect on the ecology of the Great Lakes, a few species (e.g., sea lamprey *Petromyzon marinus*, alewife *Alosa pseudoharengus*, zebra mussel *Dreissena polymorpha*, quagga mussel *D*. *bugensis*, and spiny waterflea *Bythotrephes longimanus* have had substantial negative effects (Bunnell et al. 2014; Vanderploeg et al. 2002a; b). Still others, such as the white perch *Morone americana*, have increased to relatively high abundances but their net effects on resident species have been relatively uncertain (Pothoven and Höök 2015).

In addition to these known non-indigenous species in the Great Lakes region, several other species are likely to invade the Great Lakes, including fishes (e.g., bighead carp *H*. *nobilis*, black carp, *Mylopharyngodon piceus*, silver carp *Hypophthalmichthys molitrix*, and northern snakehead *Channa argus*), macroinvertebrates (e.g., golden mussel *Limnoperna fortunei*, killer shrimp *Dikerogammarus villosus*), zooplankton (e.g., calanoid copepod *Calanipeda aquaedulcis*) and plants (e.g., Brazilian waterweed *Egeria densa*) (Davidson et al. 2017; Fusaro et al. 2016). Currently, little is known of the potential ecological and economic effects of these invaders, highlighting the need to develop predictive capabilities for discerning direction and magnitude of potential effects.

Perhaps the greatest invasion threat to the Great Lakes is the potential introduction and impact of bighead and silver carp (collectively bigheaded carps, BHC) on Great Lakes aquatic ecosystems. This threat has elicited great concerns from managers, decision makers, and the public (Kokotovich and Andow 2017). BHC were first introduced into aquaculture ponds in the southern US in 1973, escaped into the Mississippi River drainage (Cudmore et al. 2012; Freeze and Henderson 1982) and rapidly expanded their range northward (Cudmore et al. 2012; Kolar et al. 2007). Within the Mississippi and Illinois River systems, they now comprise a large fraction of the total fish biomass, and have had significant negative effects on plankton and planktivorous fishes (Irons et al. 2007; Pendleton et al. 2017; Phelps et al. 2017). Predictive risk assessment tools may inform prevention and management efforts by evaluating the potential establishment, spread, and ecosystem effects of BHC invasions to the Great Lakes (Davidson et al. 2017; Zhang et al. 2016, 2019). Specifically, these tools address questions about: (1) how likely are BHC to become established, (2) what are the potential effects (negative or positive) of these species on the ecology of the Great Lakes, and (3) what Great Lakes habitats are most susceptible to the establishment of BHC and their likely impacts.

Several studies have investigated the likelihood of BHC invasion, establishment and food-web effects in the Great Lakes (Cuddington et al. 2014; Kocovsky et al. 2012; Zhang et al. 2016). Climate regimes within the Great Lakes appear favorable to supporting BHC (Herborg et al. 2007). Sufficient spawning habitats exist within the Great Lakes basin with appropriate thermal and hydrologic characteristics required for the successful recruitment of BHC (Garcia et al. 2015; Kocovsky et al. 2012; Kolar et al. 2007). Moreover, productive habitats in nearshore areas of the Great Lakes (Cooke and Hill 2010; Herborg et al. 2007; Wittmann et al. 2017) and much of Lake Erie (Anderson et al. 2015; Zhang et al. 2016) will likely support sufficient prey biomass to sustain BHC, but open water habitats of the upper Great Lakes are likely too oligotrophic to support adequate BHC growth (Anderson et al. 2017; Cooke and Hill 2010). Cuddington et al. (2014) found that the minimum number of BHC needed to establish a population may be less than 20 sexually mature adults, but the number of fish depends upon the availability of tributaries for spawning and the ability of BHC to find them. A food web model developed to project the effects of BHC on Lake Erie suggested that if they became established in the lake, they might eventually comprise up to 34% of the total fish biomass, and have negative effects on planktivorous fishes (e.g., emerald shiner *Notropis atherinoides*) and positive effects on some piscivores (e.g., smallmouth bass *Micropterus dolomieu*) (Zhang et al. 2016). However, Zhang et al.’s (2016) application of the Ecopath with Ecosim food web model did not explicitly consider the influence of temperature on biological processes, was parameterized with average annual process rates with a monthly time step, and simulated fish species as biomass pools, which may smooth over seasonal peaks in BHC effects.

None of the prior efforts to model the effects of BHC on the food web have included size-based interactions among predators and prey that can affect early life survival and reproductive success. We believe a multi-species individual-based bioenergetics model (ms-IBM) that includes detailed interactions among individual fish, and temperature effects on fish feeding and growth on a daily time scale can inform a risk assessment of BHC impacts on a Great Lakes food web. Herein, we report on the development and use of a ms-IBM to predict the probability of BHC becoming established in Saginaw Bay given a range of BHC seed population sizes and age-0 survival rates, and then quantify their potential food-web effects once they become established in the Bay.

## Methods

### Site description

**Fig. 1:**
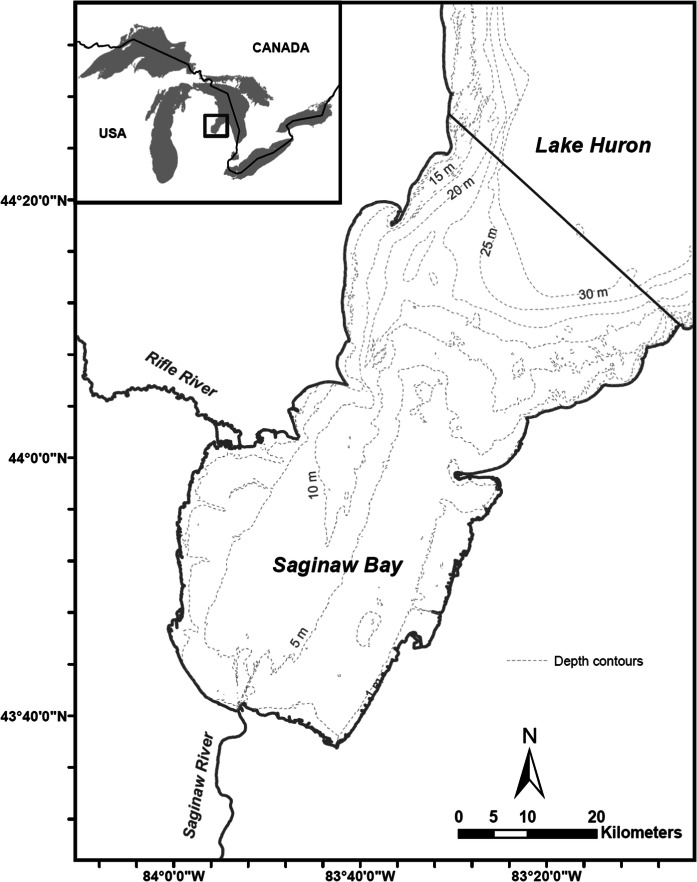
Saginaw Bay showing the two tributaries (Rifle River and Saginaw River) and Lake Huron. The four spatial habitats modeled are Saginaw Bay proper, tributaries (Rifle and Saginaw River) for spawning, and Lake Huron for out-migrating species (see text)

Saginaw Bay (Fig. 1) is a mesotrophic system with a surface area of 2960 km^2^ and an average depth of 9.5 m (Nalepa et al. 2003), with two tributaries (Saginaw and Rifle Rivers) capable of providing suitable spawning habitat for BHC (Kolar et al. 2007). The Bay currently supports valuable fisheries for walleye *Sander vitreus*, and yellow perch *Perca flavescens* (Fielder and Bence 2014; Fielder et al. 2007). The bay receives major phosphorous loads ranging from 208 to 1400 metric tonnes/year (Stow and Cha 2013) from the Saginaw River (watershed area of 22,260 km^2^), and is bordered by the oligotrophic, deeper waters of Lake Huron’s main basin. The spatial domain for the model is defined by three habitats (Saginaw Bay, tributaries to Saginaw Bay, and Lake Huron; Fig. 1). We included the open waters of Lake Huron as a model box because walleye emigrate from Saginaw Bay seasonally to Lake Huron. The model consists of 4 spatial units. Saginaw Bay is modeled as a single spatial unit, while its two tributaries are modeled two spatial units for spawning sites for fishes that spawn in rivers and Lake Huron is modeled as a single unit for holding migratory fishes that leave the bay (Fig. 1).

### Model description

**Fig. 2:**
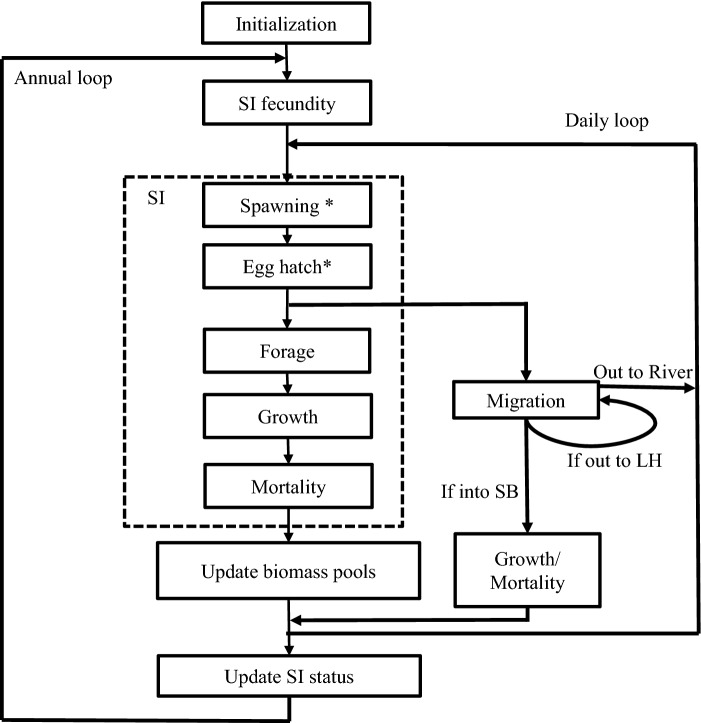
Flow chart of the individual-based community model showing daily and annual functions. Annually, an SI’s status is updated for age, maturation and migratory status. Asterisk indicates functions are carried out daily but will skip once the processes are finished for the year. ‘LH” indicates Lake Huron; ‘SB’ indicates Saginaw Bay. ‘SI’ is a model superindividual

A detailed description of the model configuration, calibration and data sources is provided in the Supplementary materials (Electronic Supplement A, B). Here we provide a summary description highlighting the major components of our modeling approach. The model is a multiple species individual-based model (ms-IBM) that tracks the daily population dynamics of six different species (populations) as super individuals (SIs) and seven different prey biomass groups over multiple generations (Fig. 2) for 50 years. The use of SIs is a computational method to simulate a large-number populations with a reduced number of representative individuals. A SI represents multiple identical individuals in the population and the same number of SIs always remain in simulations; mortality is represented by reducing the number of population individuals each SI represents (Scheffer et al. 1995). When SI reaches a specified maximum age, they are removed from the model and used to keep track of the newly produced young. Anytime the number of individuals represented by a SI drops below a predefined value, they are removed from the population. All calculations in the model involving super-individuals (e.g., SI predators eating SI prey) and model outputs (e.g., abundance) are always adjusted by the number of population individuals associated with each SI (see Rose et al. 2015 for more details).

We chose six species to model as SIs-bighead carp, silver carp, walleye, yellow perch, rainbow smelt *Osmerus mordax*, and round goby *Neogobius melanostomus*. We chose these species as SIs as their predator–prey interactions, fecundity, and prey selection are all strongly size dependent. Piscivorous walleye and omnivorous yellow perch were chosen because of their value to Saginaw Bay fisheries and food-web dynamics (Kao et al. 2014), and omnivorous rainbow smelt and benthivorous round goby were chosen because they are abundant prey fish and comprise a significant fraction of walleye diet (Pothoven et al. 2017). Prey groups modeled as biomass pools included potential prey (zooplankton, phytoplankton, detritus) for BHC, benthos prey for walleye, yellow perch and round goby, and forage fish as an alternate fish prey source for walleye and yellow perch. In addition, we modeled two invasive species groups *Bythotrephes* and *Dreissena* mussels as biomass pools because they serve as prey for planktivorous fishes and round goby, respectively, and would compete for plankton with BHC. All SI populations and biomass groups are dynamically coupled, with SI populations feeding on other SI groups and prey biomass groups, and different prey biomass groups feeding on one another or cycling organic material to different groups (Table 1).

**Table 1:** Prey–predator interactions in the simulated food web. X indicates an interaction between prey (in rows) and predators (in columns). The number IDs for predators are the same as those for prey

Prey\predator ID	1	2	3	4	5	6	7	8	9	10	11
1. Silver Carp			X	X	X	X					
2. Bighead Carp			X	X	X	X					
3. Walleye			X	X	X	X					
4. Yellow Perch			X	X	X	X					
5. Round Goby			X	X	X	X					
6. Rainbow Smelt			X	X	X	X					
7. Forage fish			X	X	X	X					
8. Benthos			X	X	X	X	X				
9. Dreissenid mussels					X						
10. Zooplankton	X	X	X	X	X	X	X				X
11. Bythotrephes	X	X	X	X	X	X					
12. Phytoplankton	X	X					X	X	X	X	
13. Detritus	X	X						X	X	X	

Model simulations cycle daily through individuals and biomass pools (Fig. 2). Each day, individuals that do not reside on spawning grounds or move outside the model domain (i.e., migratory fish) are assessed for consumption, respiration, growth, mortality, movement, and spawning. Fish that migrate to Lake Huron or to the spawning grounds are assessed daily for the time period that they are in the bay, but for the time period they are out of the bay, they are assessed once for growth and mortality when they migrate back into the bay. Maturation is assessed for all SIs annually.

### Water temperature

Water temperature was used as input for reproduction and for modeling growth rates of individual species (see below) and was determined from an empirical relationship as a function of Julian days (T. Johengen, pers.comm, Electronic Supplement A).

### Prey biomass pools

Dynamics of the prey biomass groups follow the equations used in EcoPath with EcoSim (EwE; (Christensen and Walters 2004; Pauly et al. 2000), which we modified for a daily time step (Electronic Supplement A). Inputs included initial biomass, production to biomass ratios (P/B), consumption to biomass ratios (Q/B), and diet composition (Electronic Supplement Table A3). Prey biomass values were initialized based on empirical observations from Saginaw Bay made from 1991–1996 to 2009–2011 distributed throughout Saginaw Bay (Nalepa et al. 2003; Pothoven et al. 2013; Stow and Hook 2013). Biomass pools for phytoplankton vary seasonally to represent greater production in summer and late fall using pre-defined monthly P/B ratios. In addition to predator–prey interactions among prey pools, individual fishes consumed prey pools, and the total biomass consumed is removed from the prey pool.

### Fish super individuals (SIs)

At initialization, all state variables for SIs are assigned to individuals including age from a specified distribution (Electronic Supplement Table A1), length from mean total length-at-age (Electronic Supplement Table A2), sex, spawning time, and hatching time for age-0 fish (Electronic Supplement A). The number of population individuals represented by each SI (along with initial weights from assigned lengths) for the six species were specified to match the biomass values observed in Saginaw Bay from 1998 to 2011 (Fielder and Bence 2014; Stow and Hook 2013). Ages are assigned based on literature values for observed age distributions in this system or in similar systems. Length at age is assigned from a random normal distribution using species-specific mean lengths at age and standard deviations. Sex for each SI is assigned randomly assuming a 50–50 sex ratio.

#### Maturation, reproduction and egg development

For SIs, maturation is based on either size or age, depending on species (Electronic Supplement A). BHC, walleye, and rainbow smelt spawn in tributaries, whereas yellow perch and round goby spawn in Saginaw Bay. The earliest possible date that SIs can spawn is fixed for each species each year (Electronic Supplement Table A6). The temperature at which a SI spawns is randomly assigned from a uniform distribution with temperature range varying by species (Electronic Supplement Table A6). Depending on time of year, mature individuals spawn when the environmental temperature first exceeds their assigned spawning temperature. Species that spawn in tributaries arrive when the water temperature reaches 2 °C below their assigned spawning temperature, and after spawning return to Saginaw Bay.

Fecundity for SIs is assigned using species-specific fecundity relationships modeled as a function of mass or length (Electronic Supplement Table A7). Eggs from each SI are then assigned to a new individual SI. BHC, walleye, yellow perch, and rainbow smelt eggs develop and then hatch after a required number of thermal units are accumulated (Auer 1982; Chapman and George 2011; MacInnis and Corkum 2000; Rose et al. 1999), while round goby eggs hatch 18 days after spawning (Marsden et al. 1996). At hatch, larvae are assigned species-specific hatching sizes (length and mass) drawn from a normal distribution, and temperature-dependent time (days) until first feeding (Electronic Supplement Table A8).

#### Growth

Species-specific growth rates for all life stages are determined using species-specific foraging and bioenergetics models (Hanson et al. 1997). Foraging (i.e., consumption) for all species is modeled based on a modified type II functional response and takes the form:

1 $$C_{i,j} = \frac{{Cmax_{i} Vul_{i,j} B_{j } a_{i,j} /K_{i,j} }}{{1 + \mathop \sum \nolimits_{j}^{6} Vul_{i,j} B_{j} a_{i,j} /K_{i,j} }}$$

where *Cmax*_*i*_ is the maximum consumption from the bioenergetics model of individual *i*, *Vul*_*i*,*j*_ is the vulnerability of prey species *j* to individual *i*, *B*_*j*_ is the biomass of prey *j* available to individual *i* which was updated after each simulation day, *a*_*i*,*j*_ is a parameter representing the search and encounter of prey type *j* by individual *i*, and *K*_*i*,*j*_ is the half saturation constant of fish species *i* feeding on prey *j*. Growth was modeled using the standard Wisconsin bioenergetics model (Hanson et al. 1997):

2 $$W_{i} = C_{i} - R_{i} - F_{i} - U_{i} - S_{i}$$

where *W*_*i*_ is wet weight (g), *C*_*i*_ (g g^−1^ d^−1^) is total consumption of all prey types by individual *i*, *R*_*i*_ is respiration (g g^−1^ d^−1^), *F*_*i*_ is egestion (g g^−1^ d^−1^), *U*_*i*_ (g g^−1^ d^−1^) is excretion, and *S*_*i*_ (g g^−1^ d^−1^) is specific dynamic action. For details on the foraging model, growth model, and model parameterization and calibration see Electronic Supplement A.

#### Mortality

Fish SIs experienced three types of mortality: starvation, predation, and background (other) mortality (Electronic Supplement A). Young fish begin to starve when an individual’s weight drops 50% below its expected weight given its length (Letcher et al. 1996). In general, the starvation threshold at which fish die due to starvation ranges from 58 to 87% (Letcher et al. 1996). Because we used super individuals, we chose not to remove all individuals represented by a SI at a single time step. When an individual’s weight dropped 50% below its expected weight given its length, 30% of the individuals represented by the SI were removed daily. If a SI’s weight dropped below 10% of its expected weight, all remaining individuals represented by the SI were removed. Predation mortality occurs when a SI is preyed upon by another SI. In this case, the number of individuals removed from an SI is dependent on the grams consumed by the predatory SI multiplied by how many population individuals are represented by the predator SI. Finally, to mimic background mortality, the number of population individuals represented by each SI is removed using a daily mortality rate that is a modified function of the individual’s mass (Houde 2002; Lorenzen 1996).

#### Movement

There are two types of movements performed by the SIs: spawning migrations and migrations from Saginaw Bay to Lake Huron. Spawning migrations depend on the preferred temperature and spawning habitat of individual species and are immediate movements to the spawning grounds (e.g., river). Moving to Lake Huron (and treated as migratory fish whose dynamics are not tracked daily) only occurs for migratory species (walleye and rainbow smelt) and is an immediate movement based on day of year. Walleye age 3+ have a 50% probability of migrating for spawning each year. If a walleye SI is assigned a migratory status to Lake Huron, it leaves the system on day 181 and returns on day 1 of the following year. Walleye could migrate 1 year and not the next. Age 1+ rainbow smelt are all migratory and only remain in the system around spawning times, leaving on day 151.

### Model calibration

We calibrated the model using a three-step process. Our first step was to run the model without fish SIs to capture adequate seasonal dynamics and biomass of prey pools for 2 years. We then added in model SI species without BHC and ran the model for 5 years to verify that fish growth rates and abundances were similar to observed values. To calibrate model fish size-at-age and biomass, we adjusted foraging coefficients and egg and adult stage background mortality rates (Electronic Supplement A). Finally, we introduced a population of 1000 bighead carp or 1000 silver carp into the model and simulated their growth for 5 years without changing any baseline values, and calibrated the carps’ length-at-age. The calibration results are presented in the Electronic Supplement B.

Observed data on prey biomass for model calibration were based on field surveys of Saginaw Bay conducted from 1991–1996 to 2009–2011 (Stow and Hook 2013). Size-at-age and abundance estimates for walleye and yellow perch were derived from Saginaw Bay trawl and gill net data from 1986 to 2011 (Fielder and Thomas 2006). Rainbow smelt length at age and maturity values were derived from Bailey (1964). Round goby data were derived using P/B values from Kao et al. (2014) with lengths at age from Johnson et al. (2005).

### Simulation scenarios

Baseline-scenario simulations included all species except bighead and silver carp. We ran several simulation scenarios to assess the likelihood of BHC establishment and to determine their food-web effects. Establishment was defined as achieving a stable population size of a 1000 individuals after 20 years (e.g., Cuddington et al. 2014). First, we simulated the possible establishment of BHC in Saginaw Bay for a wide range of seed population sizes: 5, 10, 20, 100, 1000, 10,000, 100,000, and 1,000,000 individual bighead carp or silver carp added into the system at initialization. We used this wide range of seed populations specifically to generate a probability curve for establishment. The lower end of the range of seed population sizes (e.g., ≤ 1000 individuals) is analogous to small numbers of fish being introduced accidentally, intentionally or through long range dispersal events from a distant site that has already been successfully invaded (e.g., Alsip et al. 2019). The upper end of the range of seed population (sizes ≥ 10,000 individuals) is, to a limited extent, analogous to a scenario where BHC have become established elsewhere in Lake Huron and that population’s wave front is moving towards Saginaw Bay (e.g., Chuang and Peterson 2016). Simulations using the upper end of the range of seed population sizes serve to give an indication of community population dynamics/effects when an invasion by BHC becomes inevitable and serves to constrain our range of possible simulations. Age structure and mean length of BHC were randomly selected from uniform distributions based on literature values for a similar latitude river in China (Electronic Supplement Tables A1, A2). Sex was randomly assigned assuming a 1:1 sex ratio. Second, we used the scenarios of 1 million bighead carp or 1 million silver carp at initialization to determine the impacts of BHC on the Saginaw Bay ecosystem. Since actual survival rate for age-0 BHC is unknown (i.e., recruitment), we ran all simulations with a range of survival rates to bound the problem: high annual survival (H; S ~ 20.6–32.2%, calculated from Lorenzen 1996) (Electronic Supplement A), intermediate annual survival (M; S ~ 0.4–2.2%), and low annual survival (L; S ~ 0.06–0.2%, an age-0 survival rate similar to walleye) (Rose et al. 1999). Model simulations were run for 50 years using a daily time step. Simulation results were exported on day 0 for every 30 days till day 360 each year. Each simulation was repeated 10 times to allow us to examine the effects of stochasticity within the model on variability in model predictions. On day 300 of the last 10 years of each simulation, we recorded the average (+ 1 SD) biomass of all prey pools, abundance and biomass of each fish species, size-at-age of each fish species, and age-specific consumption of each prey type. We computed survival of age-0 model fish as the population size on day 360 divided by the population on day 0.

## Results

### Likelihood of establishment

**Fig. 3:**
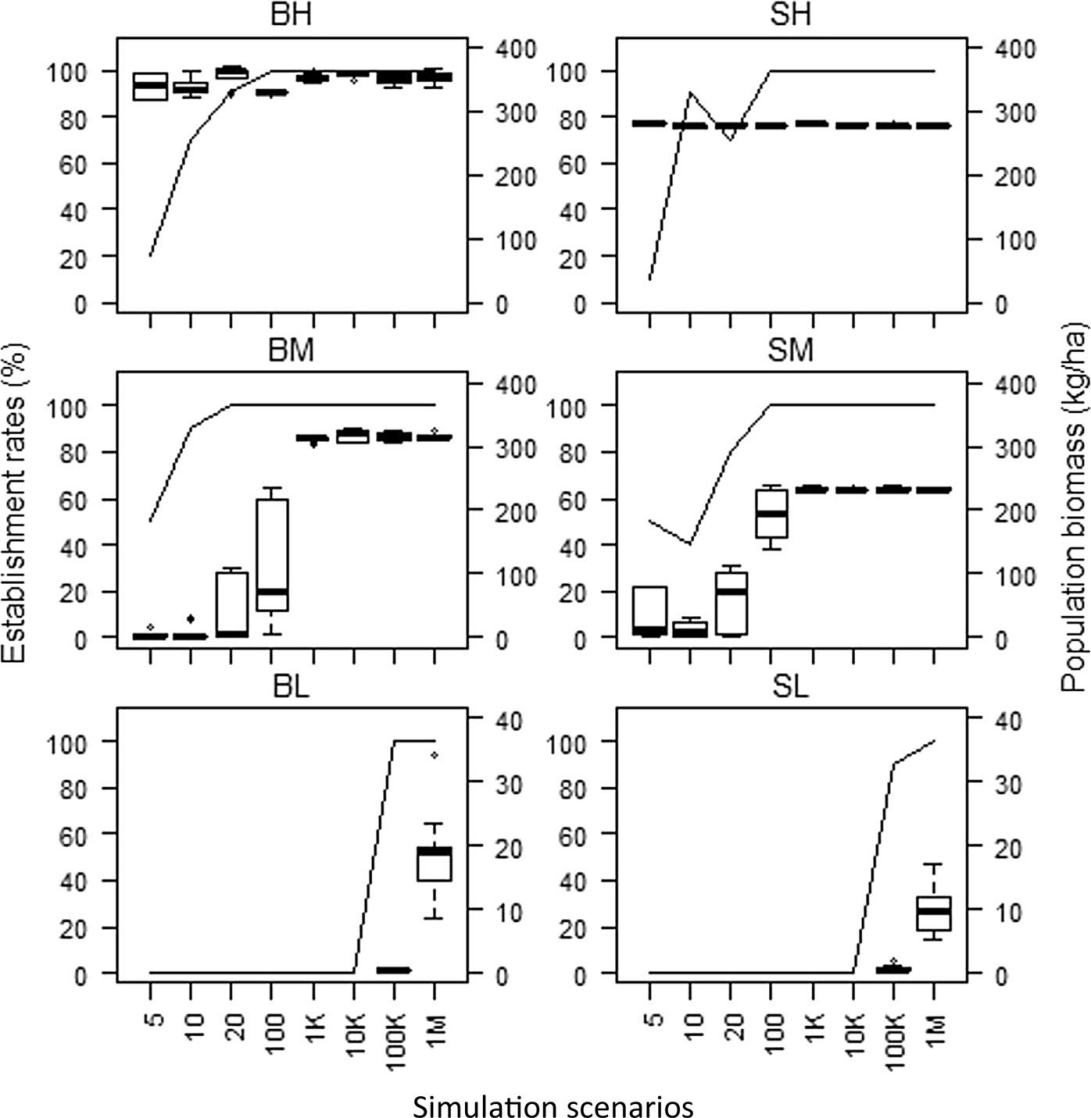
Establishment scenarios for bighead (B) and silver (S) carp under varying age-0 carp annual survival rates (H—high, M—intermediate, and L—low) and initial number of individuals (seed population). The probability of establishment was computed from 10 replicate runs of each scenario, while the population biomass (kg ha^−1^) was only based on the runs with successful establishment of each scenario. Successful establishment was defined as a stable population of more than 1000 individuals after 20 years. Population biomass was the average of total population biomass over the last 10 simulation years. Note the *y*-axis are of different scales

The likelihood of BHC establishment varied depending upon the initial number of individuals (i.e., founder size or seed population) and assumptions of age-0 carp survival (recruitment). Successful establishment for both bighead and silver carp occurred in all scenarios when age-0 survival was high, with final population biomass (averaged over the last 10 simulation years) ranging from 319.1 to 370.7 kg ha^−1^ for bighead carp, and from 272.7 to 282.1 kg ha^−1^ for silver carp (Fig. 3). For bighead carp with high age-0 survival, probability of establishment ranged from 20% with an initial population of five individuals to 100% when the initial population had 100 individuals or more. Silver carp showed a similar trend and ranged from a 10% probability of establishment at the lowest initial population size to 100% at 100 individuals or more. In contrast, under the assumption of low age-0 survival, successful establishment of BHC only occurred when the initial seed population was 100,000 individuals or higher for both species, and only achieved 100% probability of establishment with 1,000,000 individuals with a maximum population biomass of 34.1 kg ha^−1^ for bighead carp and 17.2 kg ha^−1^ for silver carp. For intermediate age-0 survival, intermediate establishment success occurred for all scenarios with final biomass ranging from 0.4 to 329.3 kg ha^−1^ for bighead carp and 0.4–238.4 kg ha^−1^ for silver carp. BHC established successfully 100% of the time when there were 20 initial individuals or more for bighead carp and 100 individuals or more for silver carp under the intermediate age-0 survival scenario. When initial population size fell below 100 individuals, established population biomass ranged from 0.4 to 109.1 kg ha^−1^ for bighead carp and from 0.4 to 114 kg ha^−1^ for silver carp.

**Fig. 4:**
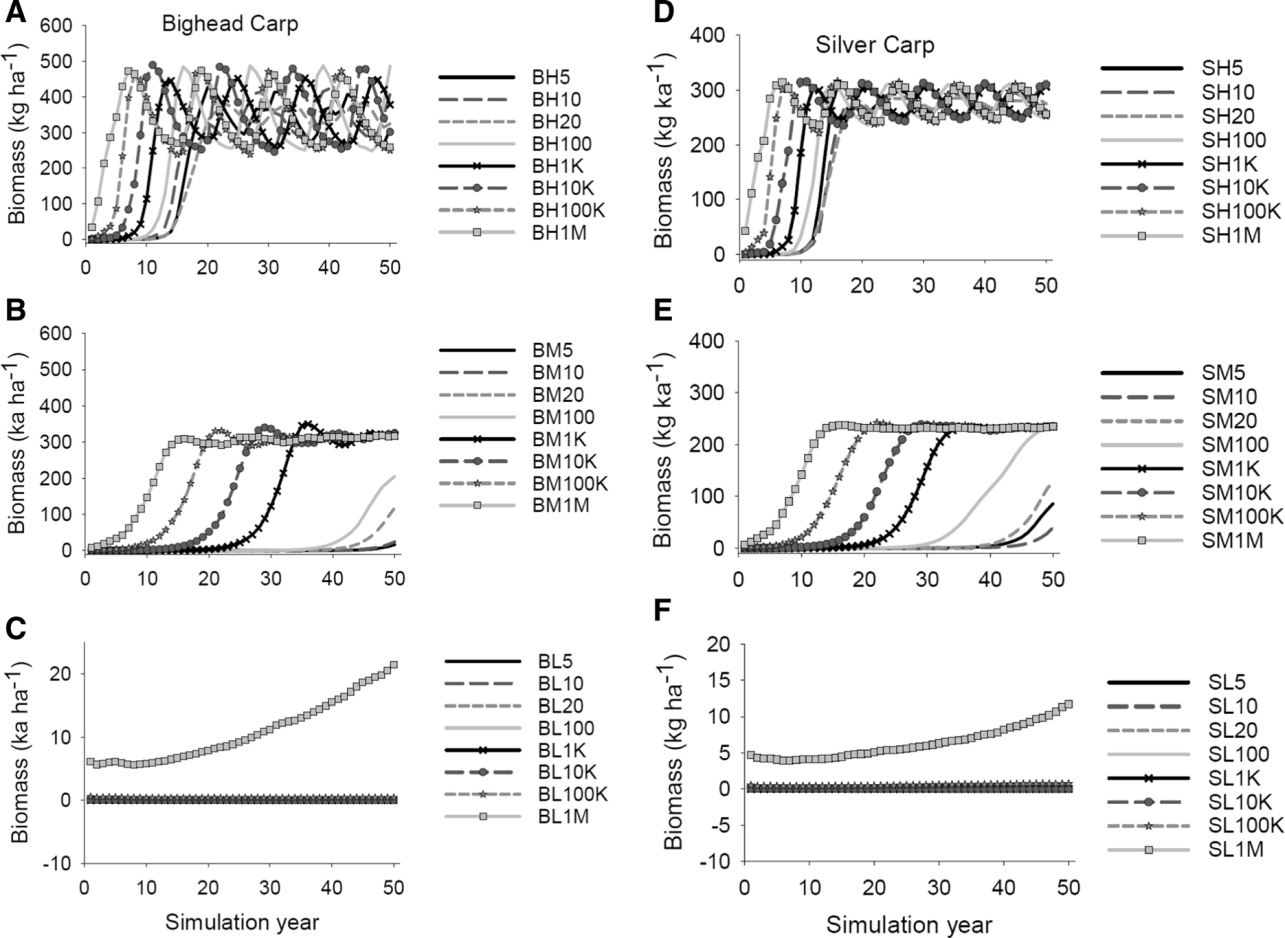
Predicted biomass of bighead (B) and silver (S) carp biomass (kg ha^−1^) from 50-year simulation runs of establishment scenarios. Only simulation runs of successful establishment were included. Initial numbers of carp introduced were: 5, 10, 20, 100, 1000, 10,000, 100,0000, and 1 million individuals of **a** bighead carp with high age-0 annual survival (H), **b** bighead carp with intermediate age-0 annual survival (M), **c** bighead carp with low age-0 annual survival (L), **d** silver carp with high age-0 annual survival, **e** silver carp with intermediate age-0 annual survival, **f** silver carp with low age-0 annual survival. Numbers in the legend represent the initial number of bigheaded carp introduced

Time to establishment and equilibrium biomass for BHC also was a function of age-0 survival rate and size of seed population. After establishment, and under the high survival scenarios, bighead carp reached a dynamic equilibrium within about 20 years and began to oscillate between 240 and 500 kg ha^−1^. Scenarios with larger seed populations tended to achieve equilibrium much more quickly (within 10 years) than the smaller seed populations (Fig. 4a). Similar to the high survival scenarios, bighead carp population biomass under an intermediate survival scenario and with larger seed populations (≥ 1000 individuals) achieved equilibrium within 15–35 years, with biomass oscillating between approximately 250–350 kg ha^−1^ (Fig. 4b). Small seed populations (5–100 individuals) failed to reach equilibrium by the end of the 50 years simulations, but had an upward trajectory at the end of the simulation run. Under the low survival scenarios and seed populations of 1 million individuals, equilibrium was not reached prior to the end of 50 simulation years, but slowly increased over the 50 years simulation and achieved a biomass of around 20 kg ha^−1^ (Fig. 4c). Silver carp had population growth patterns similar to bighead carp, but with substantially lower equilibrium biomasses under the high (230–320 kg ha^−1^), and intermediate (220–240 kg ha^−1^) survival scenarios (Fig. 4d, e). For the low survival scenarios with a seed population of one million individuals, the final biomass for silver carp was 12 kg ha^−1^ after 50 years (Fig. 4f) but was slowly increasing and did not reach a stable equilibrium by the end of the simulation.

### Food-web effects

Scenarios with 1 million individuals became established with 100% success regardless of the assumed age-0 survival rate. Thus, we chose those scenarios to study BHC impacts on the food web. The intermediate and high survival scenarios resulted in equilibrium BHC biomass > 200 kg ha^−1^, which we considered to be relatively high biomass based on BHCs biomasses observed in other river environments and their projected biomasses in Lake Erie (Zhang et al. 2016). The low survival scenarios resulted in BHC biomass < 35 kg ha^−1^, which we subjectively considered as low biomass.

#### Biomass pools

Impacts of BHC on mean annual prey biomass pools were greatest when assumed BHC age-0 survival and simulated BHC biomass were high. Average phytoplankton biomass was similar to baseline biomass values in all scenarios except those with high and intermediate age-0 survival of silver carp (Table 2; Electronic Supplement Figs. B4–B5). Under those scenarios, mean phytoplankton biomass over the last 10 simulation years decreased by up to 11.9% from a baseline scenario of no BHC. Detrital biomass increased by 5.0% from baseline levels only when bighead carp were present under high age-0 survival, and decreased by 11.5% from baseline levels when silver carp were present under high age-0 survival scenarios. Under BHC scenarios with low age-0 survival there was no change in detrital biomass from baseline values.

**Table 2:** Total mean biomass (± SE) in wet weight of prey pools for a baseline scenario of no bigheaded carp, and various carp scenarios (B-bighead carp, and S = silver carp) and age-0 carp survival rates (H-high survival, M-intermediate, and L-low survival)

Scenarios	Phytoplankton (mg L^−1^)	Detritus (mg L^−1^)	Zooplankton (mg m^2^)	Bythotrephes (× 10^−3^ mg L^−1^)	Benthos (mg m^2^)	Dreissenids (mg m^2^)	Forage fish (mg L^−1^)
Baseline	12.25 (< 0.01)	130.99 (0.03)	16.25 (0.01)	9.79 (0.35)	53.54 (0.02)	6.61 (0.01)	6.45 (0.03)
BH	12.24 (< 0.01)	137.51 (0.38)	14.50 (0.07)	12.98 (< 0.01)	54.62 (0.06)	6.36 (0.02)	7.10 (< 0.01)
BM	12.25 (< 0.01)	133.98 (0.04)	15.19 (0.01)	12.94 (< 0.01)	54.0 (0.02)	6.18 (0.02)	6.71 (0.02)
BL	12.25 (< 0.01)	130.87 (0.01)	16.31 (< 0.01)	8.81 (0.21)	53.54 (0.01)	6.64 (< 0.01)	6.29 (0.01)
SH	10.80 (0.03)	115.97 (0.24)	16.25 (0.01)	12.87 (< 0.01)	52.05 (0.04)	6.52 (< 0.01)	6.08 (0.02)
SM	11.33 (< 0.01)	119.87 (0.04)	16.20 (0.01)	12.35 (0.07)	52.79 (0.01)	6.58 (< 0.01)	6.23 (0.01)
SL	12.23 (< 0.01)	130.61 (0.01)	16.33 (0.01)	8.65 (0.16)	53.50 (0.01)	6.62 (< 0.01)	6.24 (0.02)

Results are reported from the last 10 years of a simulation. Bigheaded carp scenarios started with an initial population size of 1 million individuals

Zooplankton, benthos, and fish groups showed mixed responses to BHC establishment depending on the assumed BHC survival rate (i.e., BHC biomass). Zooplankton biomass declined by 6.5–10.8% from baseline values for bighead carp with high and intermediate age-0 survival scenarios, but did not change under low age-0 survival scenarios or under any silver carp scenario (Table 2, Electronic Supplement Figs. B4–B6). Relative to baseline, *Bythotrephes* biomass increased by 26.2–32.6% above baseline levels when BHC age-0 survival was high or intermediate, and declined by 10.0–11.6% from baseline levels when BHC age-0 survival was low. Benthic biomass remained relatively stable in the face of BHC invasion, increasing about 2% above baseline levels under high bighead carp age-0 survival and declining by 3% under high silver carp age-0 survival. Dreissenid mussel biomass also was relatively stable in the face of carp invasion, and declined by only 3.8–6.4% from baseline under high or intermediate BHC age-0 survival. Finally, forage fish biomass increased up to 10.0% under a scenario of high bighead carp age-0 survival, but declined by up to 5.8% under all other scenarios of bighead or silver carp age-0 survival.

#### SI fish abundance, size and survival

**Fig. 5:**
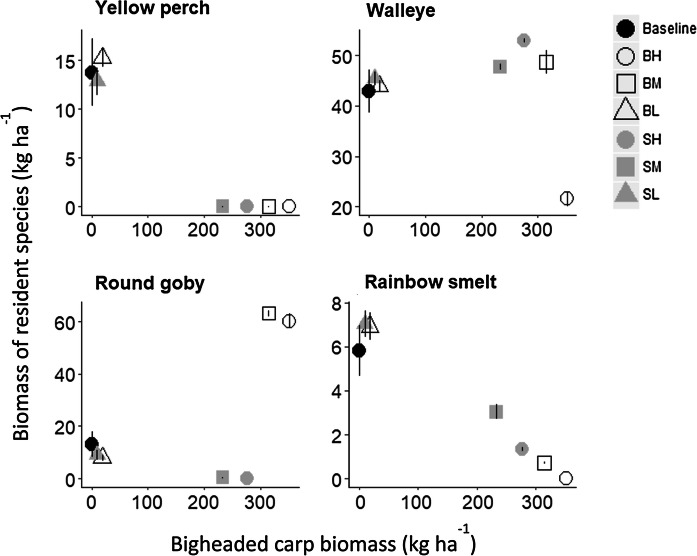
Predicted bigheaded carp biomass (kg ha^−1^) versus biomass of modeled resident species (kg ha^−1^) averaged over the last 10 simulation years of carp impact scenarios. Results are reported scenarios with initial numbers of 1 million individuals. The black dots are for baseline simulation without carp; the grey dots are for scenarios of silver carp, and the circles are for scenarios of bighead carp

When BHC biomass exceeded 200 kg ha^−1^, yellow perch were nearly extirpated (Fig. 5). Round goby abundance also collapsed under scenarios of high age-0 silver carp survival, but increased significantly at the two highest bighead carp biomasses. Rainbow smelt abundance collapsed when bighead carp biomass was high (under scenarios of high age-0 survival; Fig. 5). In general, walleye biomass ranged between 40 and 55 kg ha^−1^ as biomass increased, but declined at the highest bighead carp biomass level (Fig. 5). When walleye declined, round goby increased, suggesting that round goby abundance was negatively impacted by the increase in walleye biomass.

**Fig. 6:**
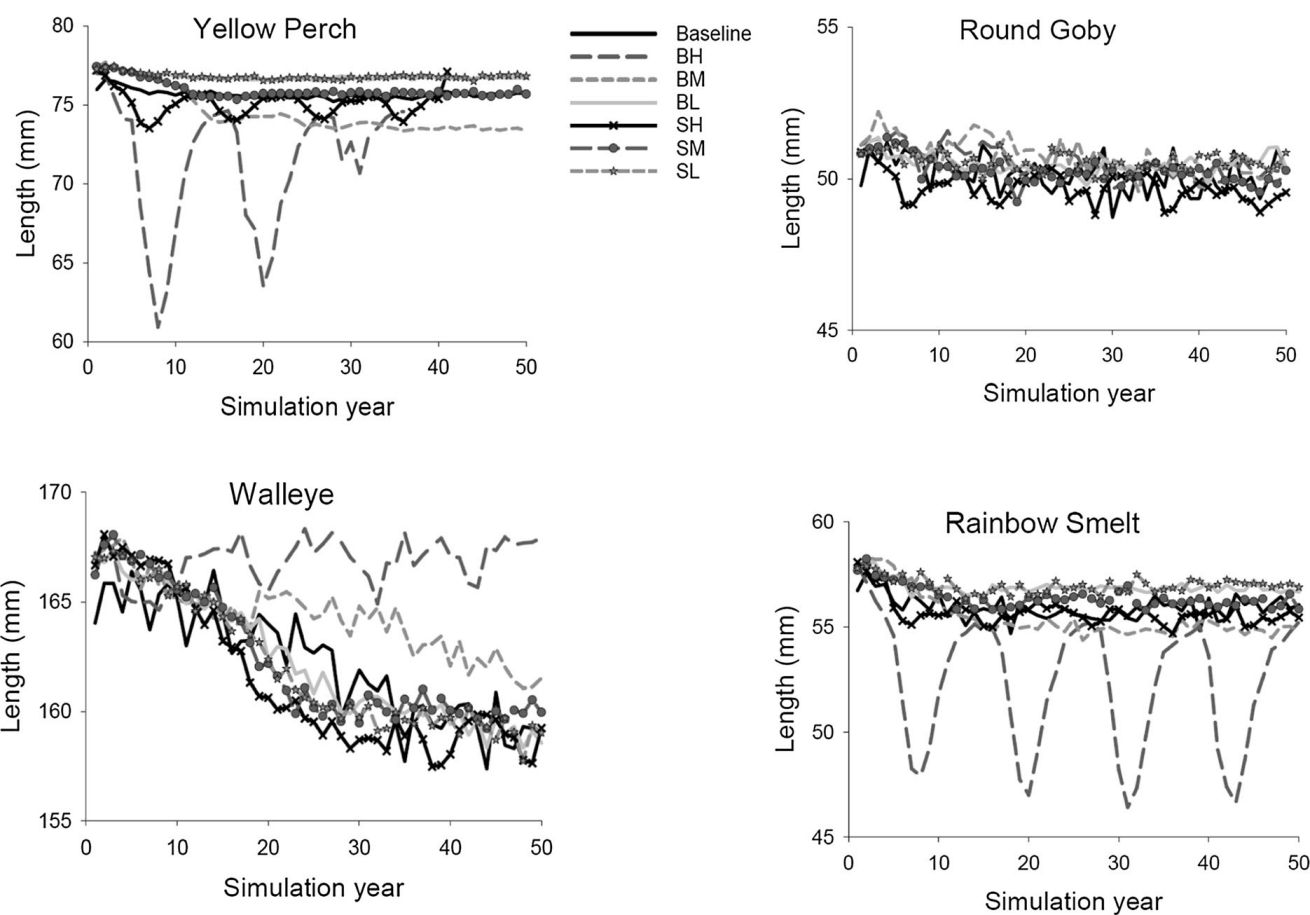
Modeled mean lengths (mm) at age-0 of **a** yellow perch, **b** walleye, **c** round goby, **d** rainbow smelt on day 300 across 50 simulation years for baseline (no bigheaded carp) and impact scenarios with initial numbers of 1 million bighead or silver carp. Baseline simulations without bigheaded carp, BH-bighead carp with high age-0 annual survival rate, BM-bighead carp with intermediate age-0 annual survival rate, BL-bighead carp with low age-0 annual survival rate, SH-silver carp with high age-0 annual survival rate, SM-silver carp with intermediate age-0 annual survival rate, and SL-silver carp with low age-0 annual survival rate

**Fig. 7:**
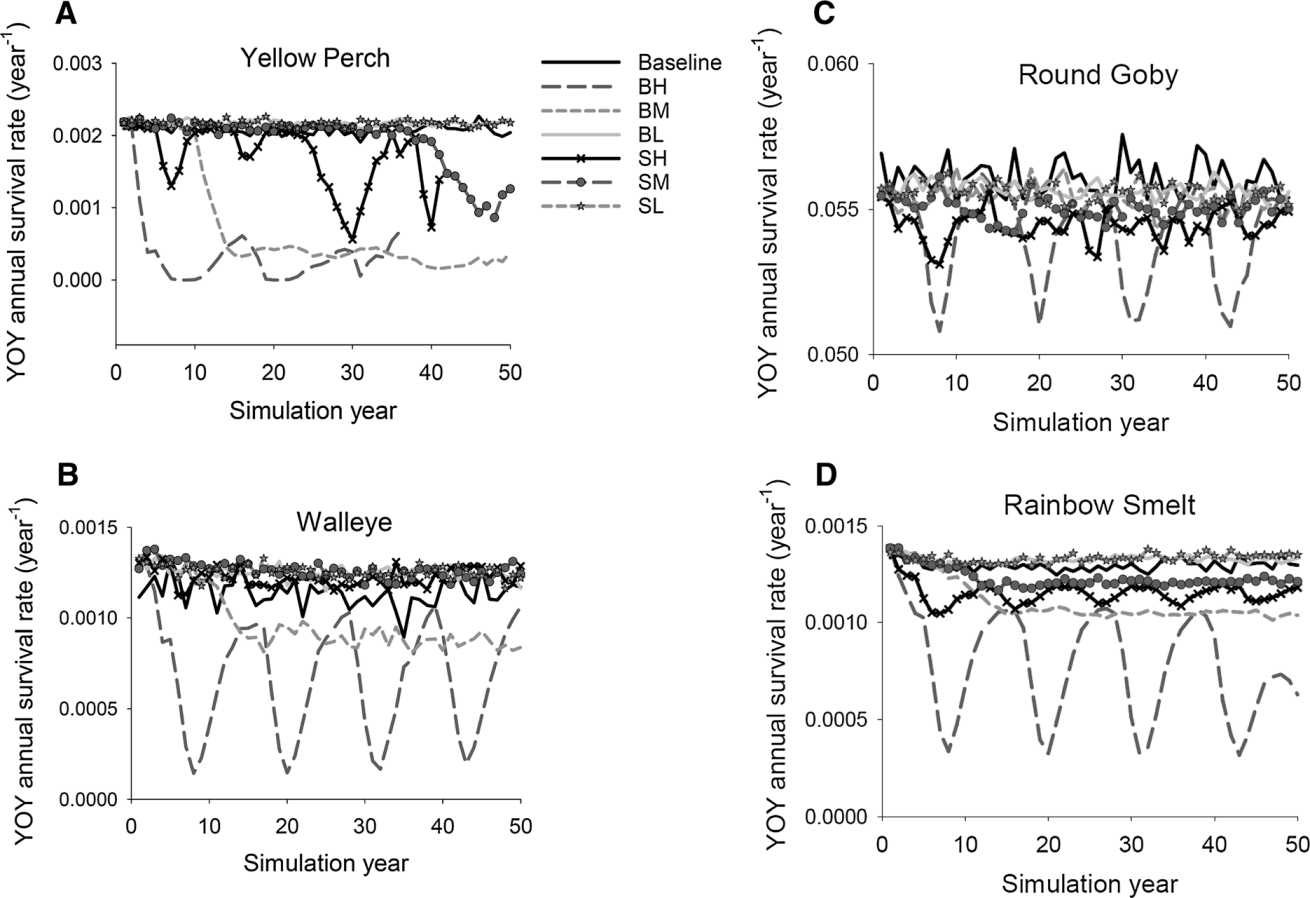
Modeled mean annual survival rates of age-0, **a** yellow perch, **b** walleye, **c** round goby, **d** rainbow smelt across 50 years of simulation for baseline (no bigheaded carp) and impact scenarios with initial numbers of 1 million bighead or silver carp. BaseL-simulations without bigheaded carp, BH-bighead carp with high age-0 annual survival rate, BM-bighead carp with intermediate age-0 annual survival rate, BL-bighead carp with low age-0 annual survival rate, SH-silver carp with high age-0 annual survival rate, SM-silver carp with intermediate age-0 annual survival rate, and SL-silver carp with low age-0 annual survival rate

To determine the cause of changes in populations when BHC became established, we examined mean length and survival of age-0 for the six species. In general, at low BHC biomass levels, mean lengths (mm) of age-0 fishes remained near baseline levels for walleye, yellow perch, rainbow smelt, and round goby (Fig. 6). However, when BHC established at high biomass levels, mean lengths of age-0 fishes declined and oscillated for yellow perch and rainbow smelt, but increased for age-0 walleye under scenarios of high bighead carp biomass. Similarly, age-0 survival of all resident fish species except walleye was generally lower than baseline during the 50-year simulation when BHC established (Fig. 7). For age-0 yellow perch under a baseline condition of no BHC, mean survival was around 0.2%, but decreased under scenarios of high and medium age-0 survival of bighead carp and high age-0 survival of silver carp. Walleye age-0 survival was 0.12–0.14% under baseline conditions of no BHC, but declined to 0.02–0.12% under scenarios of high and medium age-0 survival of bighead carp, and generally increased under all other scenarios of assumed BHC age-0 survival. Survival of age-0 round goby declined the most below baseline under the scenarios of high age-0 survival of bighead or silver carp, and less under other assumed BHC age-0 survival scenarios. Finally, survival of age-0 rainbow smelt declined the most under a scenario of high age-0 survival of bighead carp, and less so under scenarios of medium age-0 survival of bighead carp and high and medium age-0 survival of silver carp. The rapid decline in age-0 survival of each species under a scenario of high age-0 survival of bighead carp occurred within 10 simulation years.

#### Simulated impacts of BHC on fish diets

**Fig. 8:**
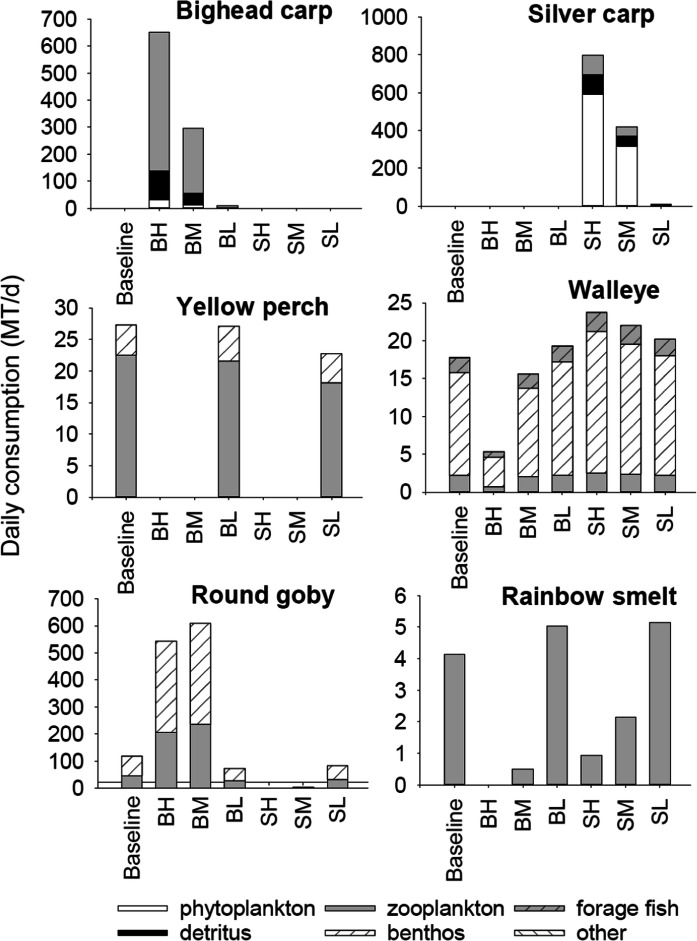
Modeled mean daily consumption (MT d^−1^) of prey by age-0 fish for the last 10 years of a 50-year impact scenario simulation with initial numbers of 1 million bighead or silver carp. Only the top three prey items were shown and the rest were grouped into “other” prey category. BaseL-simulations without bigheaded carp, BH-bighead carp with high age-0 annual survival rate, BM-bighead carp with intermediate age-0 annual survival rate, BL-bighead carp with low age-0 annual survival rate, SH-silver carp with high age-0 annual survival rate, SM-silver carp with intermediate age-0 annual survival rate, and SL-silver carp with low age-0 annual survival rate

Diets of age-0 fishes tended to be similar among simulations, but total daily consumption varied significantly depending on assumed age-0 carp survival rate and biomass of BHC (Fig. 8). Bighead carp predominantly consumed zooplankton with some detritus while silver carp consumed mostly phytoplankton followed by detritus and zooplankton (Fig. 8). Age-0 walleye consumed mostly benthos followed by forage fish and then zooplankton. Walleye consumption of benthos increased above baseline for nearly all carp scenarios but declined by 71% under a scenario resulting in high bighead carp biomass. Age-0 yellow perch consumed predominately zooplankton followed by benthos. Yellow perch consumption of zooplankton declined by 4–20% from baseline levels under scenarios of low BHC biomass, and otherwise was negligible as the yellow perch population collapsed at high BHC biomass (Fig. 5). Age-0 rainbow smelt consumption of zooplankton declined under high bighead carp biomass levels, was lower than baseline under high silver carp biomass levels, but increased above baseline levels under low silver carp biomass. Age-0 round goby consumption of benthos and zooplankton increased above baseline levels under scenarios resulting in high bighead carp biomass, was similar to baseline under low carp biomass scenarios, and was negligible under scenarios resulting in high silver carp biomass.

**Fig. 9:**
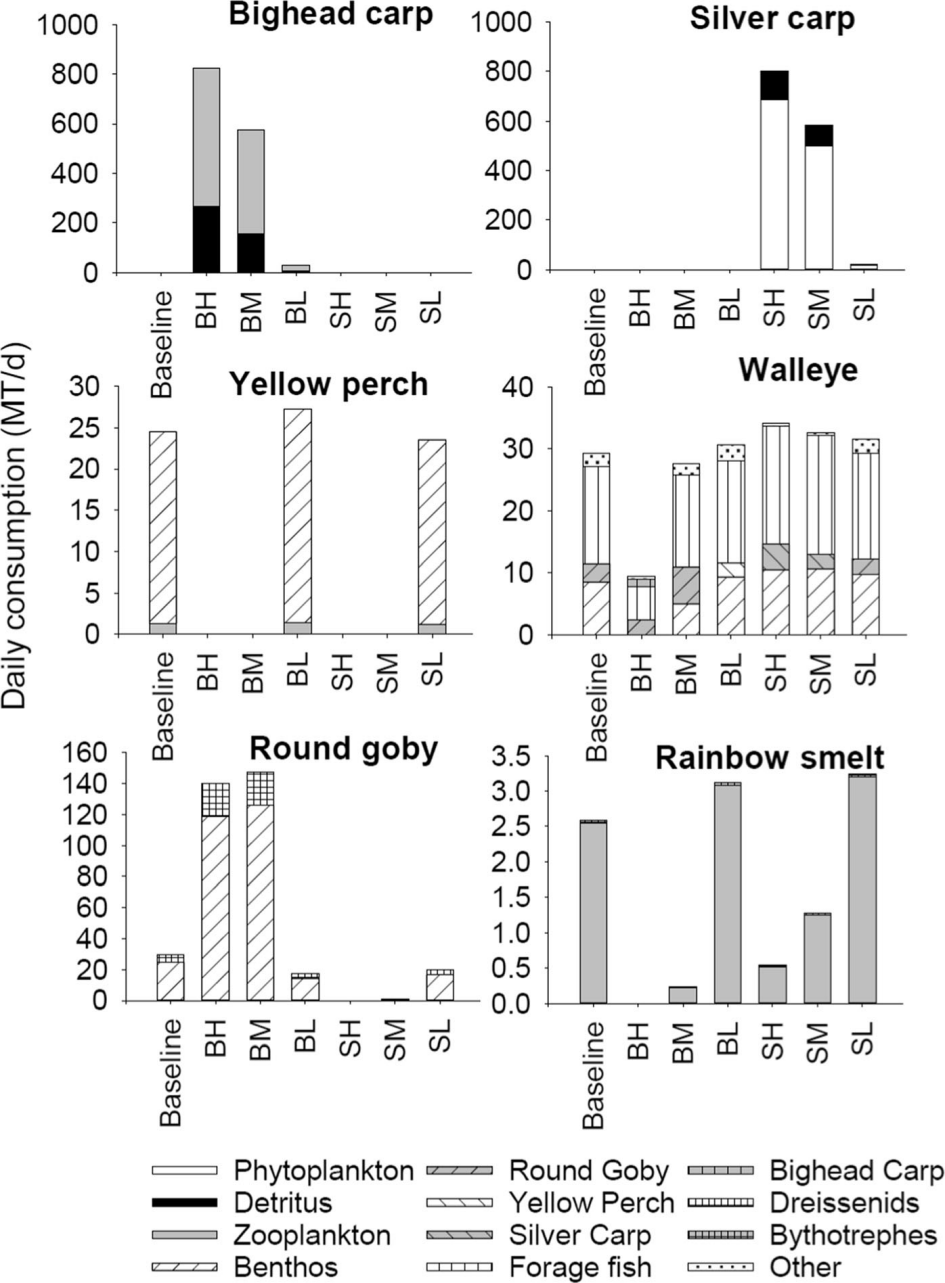
Modeled mean daily consumption (MT d^−1^) of prey by older fish (age-2 silver carp, bighead carp and yellow perch, age-3 walleye, age-1 round goby and rainbow smelt). Results are averaged for the last 10 years of a 50-year impact scenario simulation with initial numbers of 1 million bighead or silver carp. Only the top three prey items were shown and the rest were grouped into “other” prey category. BaseL-simulations without bigheaded carp, BH-bighead carp with high age-0 annual survival rate, BM-bighead carp with intermediate age-0 annual survival rate, BL-bighead carp with low age-0 annual survival rate, SH-silver carp with high age-0 annual survival rate, SM-silver carp with intermediate age-0 annual survival rate, and SL-silver carp with low age-0 annual survival rate

Although age-2 bighead and silver carp ate similar prey as age-0 BHC, prey consumption by older life stages of resident model fish responded differently to BHC biomass compared to age-0 fish. Zooplankton and then detritus dominated age-2 bighead carp diet, while phytoplankton and a small proportion of detritus (Fig. 9) dominated silver carp diet. Adult walleye had a diverse diet that included forage fish, benthos, round goby, and other prey. Walleye total consumption decreased by 6–68% under scenarios with medium and high age-0 bighead carp survival, respectively, but otherwise increased by 4–16% above baseline in response to other survival scenarios. Yellow perch predominately consumed benthos, and their population level consumption rate for benthos increased by 11% under low bighead carp biomass and decreased by 4% under low silver carp biomass. There was negligible consumption of prey by yellow perch after its population collapsed (Fig. 5). Rainbow smelt consumption of zooplankton collapsed or decreased by 51–91% under scenarios of high BHC biomass, but otherwise increased by 21–26% above baseline levels. Round goby consumption of benthos increased by fourfold when bighead carp biomass was high, but otherwise was similar to baseline levels. When silver carp biomass was high, round goby populations collapsed and their consumption of benthos declined sharply (Fig. 9).

## Discussion

### Likelihood of establishment

Likelihood of BHC establishing in Saginaw Bay increased with increasing seed population size and increasing survival rates of age-0 carp. Assuming a high age-0 survival, both bighead and silver carp had a > 50% probability of establishing with only 10 individuals. When age-0 survival was low, an initial population of 100,000 individuals was required for establishment. Similarly, Cuddington et al. (2014), using a matrix model, demonstrated that it was possible for BHC to become established, defined as a population > 1000 fish after 20 years, following a single introduction of just 10 fish. They also found that the successful establishment of BHC was sensitive to assumptions of juvenile survival rate, age at maturity, the number of rivers available for spawning and the likelihood of finding these rivers. In our model we addressed the sensitivity of early survival on establishment, but we assumed a 50% probability that BHC would find a river with suitable habitat for spawning. The ability of BHC to locate adequate spawning tributaries is unknown and would affect the probability of establishment. Furthermore, Kolar et al. (2007) identified two rivers in Saginaw Bay that would be suitable for BHC reproduction, but the actual number of suitable spawning environments may increase with more detailed studies on flow velocity and temperature conditions required to initiate BHC spawning, egg and larval drift/survival, and larval settlement (Garcia et al. 2015).

### Modeled biomass

Modeled biomass levels for BHC in Saginaw Bay were higher than those projected for Lake Erie (Zhang et al. 2016) or observed in the Illinois River (Tsehaye et al. 2013). In our simulations, BHC biomass ranged from 14.0 to 470.9 kg ha^−1^ while those projected for Lake Erie using an Ecopath with Ecosim model ranged from 52 to 104 kg ha^−1^ with a maximum value of 394 kg ha^−1^ (Zhang et al. 2016). Observed BHC biomass in the Illinois River has ranged from 190 to 250 kg ha^−1^ (Tsehaye et al. 2013). However, our projected biomass values for Saginaw Bay were lower than for areas in Asia, Europe and Israel where BHC have been introduced and have exceeded 1000 kg ha^−1^ (Costa-Pierce 1992). Overall, our model projections of maximum bighead carp biomass (471 kg ha^−1^) would represent approximately 73% of the total fish biomass estimated for Saginaw Bay in 1990 (Kao et al. 2014). This projected proportion of total fish biomass differs from that predicted for Lake Erie, where 33% of the total fish biomass (Zhang et al. 2016) could be comprised of BHC, but is consistent with estimates of 63.5% of the total fish biomass before targeted control (harvest) was implemented in some locations in the Mississippi River and Illinois River (Garvey et al. 2012, 2015). The higher contribution of BHC to Saginaw Bay’s fish biomass projected by our model compared to the BHC contribution to Lake Erie’s fish biomass may be a consequence of differences in model type and configuration. Our individual-based model of BHC in Saginaw Bay tracks energetics, growth and survival of multiple populations of individuals, and has a limited number of species and prey biomass pools, which may permit virtual BHC to reach higher biomass levels than in the Zhang et al. (2016) Lake Erie Ecopath with Ecosim model, which simulated dynamics of 47 model groups, including several groups of piscivorous fish and birds, and does not simulate the effects of temperature on growth. Moreover, the Lake Erie model represented average prey and predator biomass conditions across the whole lake, including the central and eastern basins which are less productive than the western basin. Had we simulated BHC biomass contribution to a habitat that included both Saginaw Bay and the main basin of Lake Huron, the BHC biomass contribution would have been lower.

### BHC effects on lower food web

Recent studies of BHC effects on lower trophic levels indicate their impacts may vary with prey type, habitat type, and BHC density. For example, in the Kentucky River Reservoir, significant reductions in chlorophyll a levels were found after silver carp invaded, while densities of cladocerans and copepods, and the concentration of soluble reactive phosphorus did not change from pre-invasion levels (Tumolo and Flinn 2017). However, DeBoer et al. (2018) observed declines in phytoplankton and zooplankton densities in the main channel and side channel habitats of the Illinois River after the establishment of silver carp. In the Illinois River, zooplankton assemblages shifted from a cladoceran-dominated to a rotifer-dominated system as the biomass of cladocerans, copepods and total zooplankton declined (Garvey et al. 2012; Sass et al. 2014). Although bighead and silver carp were more likely to consume rotifers than other zooplankton taxa, Sampson et al. (2009) noted a positive relationship between BHC and rotifers and a negative relationship between BHC relative abundance and cladoceran density. This pattern could be due to rotifers being released from predation and competition with the larger crustacean zooplankton. Simulations of the effects of a BHC invasion on Lake Erie’s food web suggest plankton densities would only be reduced at high BHC biomass levels (Zhang et al. 2016). Declines in the densities of cladocerans, copepods and their zooplankton predators were noted, while the densities of phytoplankton, rotifers and protozoans increased. Further, Zhang et al. (2016) explored the implications of diet flexibility for BHC, and found that if BHC consumed high levels of detritus, the impacts on phytoplankton and zooplankton would be lower.

Our IBM simulations revealed that phytoplankton and zooplankton biomass were most impacted at high BHC biomass levels. However, we were unable to tease apart their impacts on various zooplankton groups because we modeled just two zooplankton taxa (*Bythotrephes*, and all other zooplankton). In our simulations, *Bythotrephes* showed either no change or increased in biomass after BHC introduction which likely resulted from the low preference values for *Bythotrephes* that we assigned to bighead and silver carp. Overall consumptive effects of BHC on zooplankton appeared to be mediated by the lower affinity of BHC for detritus and *Bythotrephes*. However, it is unknown if BHC will eat *Bythotrephes* when other prey sources are low; as such, their impacts on this zooplankton group may be greater than shown here.

It seems reasonable to suspect that BHC could indirectly affect benthos by reducing delivery of phytoplankton and organic detritus to the lake bottom, however few studies have demonstrated that BHC can affect biomass of benthic organisms. Zhang et al. (2016) also found little impact of modeled BHC on benthic groups in Lake Erie. Here, we also found little response of benthos biomass to simulation scenarios of BHC despite declines in phytoplankton and detritus at high BHC biomass levels.

### Impacts on fishes

BHC are known to compete with planktivorous fishes for food. In laboratory studies, bighead carp have been shown to negatively impact the growth of age-0 paddlefish (*Polyodon spathula*) (Schrank et al. 2003). Similarly, long-term monitoring of the Mississippi and Illinois Rivers has revealed that abundance and condition of bigmouth buffalo *Ictiobus cyprinellus* and gizzard shad *Dorosoma cepedianum* decreased in the presence of BHC (Garvey et al. 2012; Irons et al. 2007; Pendleton et al. 2017; Phelps et al. 2017). In our simulations, the forage fish biomass pool responded positively to BHC, which increased at high bighead carp biomass, likely in response to reductions in walleye. However, rainbow smelt, the only facultative planktivore species modeled as individuals, declined with increased BHC biomass, likely driven by poor growth and survival of age-0 smelt resulting from competition with BHC for zooplankton prey. Zhang et al. (2016) also found that modeled invasion of Lake Erie by BHC negatively affected the density of planktivorous species (emerald shiner, rainbow smelt) in Lake Erie. Our results, along with those of other studies (Pendleton et al. 2017; Phelps et al. 2017; Zhang et al. 2016) suggest that BHC will have negative impacts on planktivorous fishes species within Saginaw Bay.

BHC also can reduce the biomass of piscivorous or omnivorous species if those species have a planktivorous early life history stage. In the LaGrange reach of the Illinois River, piscivorous and omnivorous fishes declined after the establishment of BHC (Garvey et al. 2012). In the Upper Mississippi River, Solomon et al. (2016) used long-term monitoring of pre- and post-BHC invasions and showed reductions in piscivorous, omnivorous, and benthivorous fishes, with a pelagic plantivorous early life history stage. In our model, reductions in yellow perch abundance were large for scenarios where BHC biomass was high. Diets of age-0 yellow perch were dominated by zooplankton, leading to significant diet overlaps and prey competition with BHC. Rapid increases in BHC biomass also resulted in declines in age-0 yellow perch length and age-0 survival. Our projections of negative impacts of BHC on yellow perch in Saginaw Bay occurred when BHC biomass reached intermediate (230–315 kg ha^−1^) or high levels (276–352 kg ha^−1^), but not much change was predicted in simulations when BHC biomass was low (≤ 18 kg ha^−1^). In a similar modeling study in Lake Erie, Zhang et al. (2016) found that when biomass of BHC exceeded 200 kg ha^−1^, yellow perch biomass declined but increased when BHC biomass was low. Similarly, DeBoer et al. (2018) observed increases in some age-0 fishes and no change in overall adult fish biomass after invasion of silver carp in the Illinois River. However, the authors were unable to explain why such increases were observed but they did suggest a few possible explanations, including predator swamping, when predators switch to feed on abundant age-0 silver carp instead of native fishes. Future work is required to determine selectivity for, and inclusion of age-0 BHC in predator diets, as this will determine the magnitude of impacts BHC will have on the ecosystem and their likelihood of establishment in the Great lakes.

Establishment of BHC also resulted in positive effects for some species. For example, in the Upper Mississippi River, shortnose gar *Lepisosteus platostomus*, green sunfish *Lepomis cyanellus*, emerald shiner, and grass carp *Ctenopharyngodon idella* all increased in abundance after the introduction of BHC (Solomon et al. 2016). In our model simulations, walleye increased at all biomass levels of bighead carp except at the highest modeled BHC biomass and remained either unchanged or increased with the presence of silver carp. In contrast to the zooplankton diet of age-0 yellow perch, walleye age-0 diet was dominated by benthos, resulting in little diet overlap with BHC, and likely explaining why walleye was not negatively impacted in our model. Similar to studies by Solomon et al. (2016), we suggest that age-0 walleye’s quick transition away from feeding on zooplankton as larvae to feeding on benthos and fish as juveniles reduced any potential competition with BHC. But, walleye were negatively impacted when bighead carp biomass was highest. Reductions in the biomass of zooplankton prey available to walleye larvae shortly after they hatch may have lowered larval growth and survival, and possibly led to lower abundances of adult walleye. This scenario is most likely to occur when bighead carp, not silver carp, are very abundant owing to bighead carp’s higher consumption of zooplankton than silver carp. However in Lake Erie, Zhang et al. (2016) found that modeled walleye were less negatively impacted than yellow perch at extremely high BHC biomass levels. These model results suggests that competition from BHC for zooplankton prey had a greater negative effect on yellow perch than on walleye. In Zhang et al.’s (2016) model, when BHC were allowed to consume larval walleye and yellow perch, both percid species declined. In our model, BHC also were allowed to consume larval yellow perch and walleye, but BHC consumption was not high enough to cause percid population declines.

It is possible that the zooplankton community composition could buffer fishes from negative impacts of BHC. For example, when comparing diets of BHC and three native filter feeders in the Mississippi River, Sampson et al. (2009) found significant diet overlap between BHC, bigmouth buffalo and gizzard shad, but not between BHC and paddlefish. This result contradicts results from Schrank et al. (2003), who found significant diet overlap between BHC and paddlefish. Sampson et al. (2009) hypothesized that differences in the zooplankton community were the reasons for this discrepancy; namely when rotifer biomass is low, BHC may switch to consuming larger zooplankton and have a greater diet overlap with paddlefish. Our model configuration included only one zooplankton prey group, forcing all fish to compete with BHC for zooplankton prey. If we had included a more diverse zooplankton community in our model, we may have found that some planktivorous fish species could be buffered from direct competition with BHC.

The importance of detritus as food for BHC may vary with plankton availability. In our model, while detritus was an important component of the diet, zooplankton and phytoplankton biomass were more important prey items for bighead and silver and carp, respectively. However, detritus maybe an important source of energy for BHC in some environments (Chen 1982; Costa-Pierce 1992; Zhang et al. 2016). Further, detritus may buffer planktivorous fishes from the negative impacts of BHC. Zhang et al. (2016) found that walleye and yellow perch were buffered from simulated competition with BHC for zooplankton when carp included a higher proportion of detritus in their diet. In our modeling studies, detritus comprised a relatively low proportion of either bighead or silver diets. These results suggest that if BHC’s preference for and consumption of detritus were to increase, planktivorous yellow perch and rainbow smelt may be less harmed by the presence of BHC.

### Modeling assumptions

We made several assumptions in configuring the IBM community model that likely affected our results. First, we assumed BHC will find suitable spawning habitats and reproduce in Saginaw Bay, Lake Huron. It is unknown how likely BHC will find rivers with suitable spawning habitat in the Bay. Clearly, lowering this probability would lower the abundance of BHC in the system, while increasing it may or may not increase the population levels depending on the degree of density dependent feedback. Also, we tested three assumed survival rates for age-0 BHC that resulted in drastically different outcomes. At high levels of age-0 BHC survival, BHC established at high biomass, resulting in a population collapse of yellow perch, while at low age-0 BHC survival, BHC biomass remained low and resident fish were unaffected. Better projections of BHC impacts on Great Lakes ecosystems will require further study of rates and mechanisms underlying reproductive behavior, first year survival and recruitment of BHC.

We also may have artificially inflated the population size and impact of BHC by our assumptions of BHC movement and also by the way we simulated prey biomass dynamics. We assumed that BHC would reside within the confines of Saginaw Bay and not emigrate to other areas of Lake Huron, thereby possibly biasing their population size and impact. Although other areas of Lake Huron have suitable spawning habitats, their productivity levels are extremely low (Bunnell et al. 2014), and it is doubtful BHC would remain there. We simulated dynamics of few interacting prey groups, making it possible that resident fish competition with BHC was higher for prey in our model environment than if we had included additional prey groups. As mentioned above, with more prey groups available to BHC, some zooplankton taxa (i.e., rotifers) may have increased when released from predation by other taxa (i.e., copepods) that may experience higher predation mortality from BHC consumption. We also incorporated a reserve for each biomass pool that could not be utilized by other groups (prey biomass pools or individual fish species) as our prey pools were general rather than specific to individual prey species. This made it impossible for these prey pools to collapse during the model run. It is possible that restricting the amount of biomass available from each prey pool to BHC may have enhanced their modeled population size, as density-dependent effects on growth and abundance may have started earlier without such reserves.

We assumed age-0 BHC would be preyed upon by piscivores identified in a structured expert judgement process (Wittmann et al. 2015; Zhang et al. 2016) and limited field and experimental studies (Anderson 2016; Sanft et al. 2018), but in reality we have only a limited understanding of predator–prey interactions between BHC and native species and thus encourage work on this topic. Studies of predation on young BHC in the Mississippi River drainage are useful (Anderson 2016; Sanft 2015; Wolf and Phelps 2017) but not sufficient to project the potential for Great Lakes piscivores to adapt to a new prey source and control or slow BHC population growth and expansion.

Finally, we chose to model one-time introductions of BHC for the establishment scenarios. It is likely that introductions of BHC will occur more than once, increasing the probability of their establishment within the bay. Our highest number of BHC introduced was equivalent to the equilibrium biomass level of yellow perch, and lower than either walleye, gizzard shad or white perch (Kao et al. 2014). We purposely used the high introduction levels to generate the probability of establishment curves. Future modeling efforts should examine the importance of multiple introductions to the projected likelihood of establishment of BHC.

## Conclusions and future work

We conclude that BHC may establish in Saginaw Bay, Lake Huron if their age-0 survival is high or if age-0 survival is intermediate and combined with a sufficiently large seed population (bighead carp > 20 individuals; silver carp > 1000 individuals), and could have a major negative impacts on resident planktivore populations, in particular yellow perch which supports an important recreational fishery (Haas and Schaefer 1992; Ivan et al. 2011), but may increase walleye populations. Modeling potential BHC establishment and food-web effects in other Great Lakes habitats will better inform risk assessment and efforts to prevent BHC introductions. For example, although many of our model findings on planktivorous fishes are similar to those reported in an earlier BHC risk assessment (Cudmore et al. 2012), we found positive effects of BHC on walleye that are contrary to those of Cudmore et al. (2012). They predicted that planktivorous fishes in nearshore habitats would experience declining growth, survival and recruitment, and assumed that piscivorous walleye also would decline in growth and abundance. However, Cudmore et al. (2012) considered and dismissed walleye predation on age-0 as BHC may quickly outgrow the gape size of most native fish predators, and the spatial distributions of juvenile BHC may not overlap with walleye. To improve projections by this model and others, we encourage future research to accurately estimate survival of age-0 BHC, assess preferences of BHC for diverse plankton assemblages, and preference of native fish to feed on young BHC (Anderson et al. 2016; Kramer et al. 2019; Wolf and Phelps 2017). In addition, the ability of BHC to locate suitable spawning habitats in new environments is unknown but is critical to quantifying establishment success and effort should be made to address how BHC may locate suitable spawning tributaries.

## Electronic supplementary material

Below is the link to the electronic supplementary material.

Supplementary material 1 (DOCX 2438 kb)
