# Supplementary material for: Potential establishment and ecological effects of bighead and silver carp in a productive embayment of the Laurentian Great Lakes
Source: Biol Invasions. 2020 Apr 21;22(8):2473–95. doi: 10.1007/s10530-020-02263-z (PMC7319282; doi:10.1007/s10530-020-02263-z)
Supplement: Supplementary file 1 — Supplementary material 1 (DOCX 2438 kb) [file 10530_2020_2263_MOESM1_ESM.docx]

Potential Establishment and Ecological Effects of Bighead and Silver Carp in a Productive Embayment of the Laurentian Great Lakes

Supplemental Material

Lori N. Ivan, Doran M. Mason, Hongyan Zhang, Edward S. Rutherford, Tim Hunter, Shaye Sable, Aaron T. Adamack, Kenneth Rose

**Supplement A: Detailed Methods**

**Overall Model Description**

We modified an individual-based community model, which was adapted from a six-species IBM code originally developed by S. Sable and K. Rose. Here we provide a detailed description of our modeling approach. The model tracks the daily population dynamics of six different species (populations) as super individuals (SI, e.g., Scheffer et al. 1995) and seven different prey biomass groups over multiple generations (Figure 2 in main text) for 50 years. The six species modeled as super individuals are Bighead Carp *Hypothalmichthys nobilis*, Silver Carp *H. molit*, Walleye *Sander vitreus*, Yellow Perch *Perca flavescens*, Rainbow Smelt *Osmerus mordax*, and Round Goby *Neogobius melanostomus*. We simulated piscivorous Walleye and omnivorous Yellow Perch as SIs because of their value to Saginaw Bay fisheries and food web dynamics (Kao et al. 2014), and Rainbow Smelt and benthivorous Round Gobv because they are abundant prey fish and comprise a significant fraction of Walleye diet. Prey groups modeled as biomass pools included potential plankton prey (phytoplankton, zooplankton, detritus) for bigheaded carp and Rainbow Smelt; benthos prey for Walleye, Yellow Perch and Round Goby; and forage fish as an alternate fish prey source for Walleye and Yellow Perch. In addition, we modeled two invasive species (*Dreissena* mussels and Spiny Waterflea *Bythotrephes longimanus*) as biomass pools because they serve as prey for Round Goby and planktivorous model fish respectively, and would compete for plankton with bigheaded carp. All SI populations and biomass groups are dynamically coupled. SI populations feed on other SIs and prey biomass groups, and different prey biomass groups feed on one another or cycle organic material to different groups (Table 1 in main text).

Three habitat types (Saginaw Bay proper, tributaries to Saginaw Bay, and Lake Huron proper; Figure 1 in main text) define the spatial domain for the model. Saginaw Bay is modeled as a single spatial unit, while two tributaries are modeled as spawning sites and Lake Huron as a boundary holding cell where migrating fish stay when they leave the bay.

Model simulations cycle daily through individuals and biomass pools (Figure 2 in main text). Each day, individuals that are not residing on spawning grounds or move outside the model domain (i.e., migratory fish) forage, respire and grow, and are assessed for growth, mortality, movement, and spawning. Fish that migrate to Lake Huron proper or to the spawning grounds are assessed daily for the time period that they are in the bay, but for the time period they are out of the bay, they are assessed once for growth and mortality when they migrate back into the bay. Maturation is assessed for all SIs annually. SIs that no longer represent any individuals are removed at the end of the model day.

Dynamics of the biomass groups follow the equations used in EcoPath with EcoSim (EwE; Christensen and Walters 2004; Pauly et al. 2000), which we modified for a daily time step. Inputs included initial biomass, production to biomass ratios (P/B), consumption to biomass ratios (Q/B), and diet composition. Biomass pools for phytoplankton vary seasonally to represent greater production in summer and late fall using predefined monthly P/B ratios. In addition to predator-prey interactions among prey pools, prey pools are consumed by individual fishes, and the total biomass consumed is then removed.

**Model initialization**

Fish modeled as super individuals were initialized at the beginning of every scenario simulation with initial population age structures and body lengths (TL, mm) of individuals at different ages (Tables A1, A2).

**Table A1**. Initialization of age structures for the super individual fish populations as a cumulative proportional age composition. Yellow Perch and Walleye ages were derived from Saginaw Bay trawl and gill net data (Fielder and Thomas 2006). Round Goby data were derived from P/B values from Kao et al. (2014). Rainbow Smelt data were derived from Bailey (1964). Silver and Bighead Carp age composition data were derived from estimated P/B values.

| Age | Silver Carp^1^ | Bighead Carp^1^ | Yellow Perch^2^ | Walleye^2^ | Round Goby^3^ | Rainbow Smelt^4^ |
| --- | --- | --- | --- | --- | --- | --- |
| Initial Proportion By Age | | | | | | |
| 1 | 0.5 | 0.5 | 0.3 | 0.4 | 0.91 | 0.29 |
| 2 | 0.65 | 0.65 | 0.57 | 0.57 | 0.99 | 0.61 |
| 3 | 0.7 | 0.7 | 0.82 | 0.66 | 1 | 0.78 |
| 4 | 0.8 | 0.8 | 0.94 | 0.75 |  | 0.93 |
| 5 | 0.85 | 0.85 | 0.98 | 0.82 |  | 0.99 |
| 6 | 0.95 | 0.95 | 1 | 0.86 |  | 1 |
| 7 | 1 | 1 |  | 0.91 |  |  |
| 8 |  |  |  | 0.94 |  |  |
| 9 |  |  |  | 0.97 |  |  |
| 10 |  |  |  | 0.98 |  |  |
| 11 |  |  |  | 0.99 |  |  |
| 12 |  |  |  | 1 |  |  |

^1^ Yan and Shi (1995); Zhang et al. (2016)

^2^ Fielder and Thomas (2006)

^3^ Kao et al. (2014)

^4^ Bailey (1964)

**Table A2**. Initialization of mean lengths (TL, mm, ± 1 S.D.) at age for the super individual fish populations. Yellow Perch and Walleye lengths were derived from Saginaw Bay trawl and gill net data (Fielder and Thomas 2006). Round Goby lengths were derived from Johnson et al. (2005). Rainbow Smelt data were derived from Bailey (1964). Silver and Bighead Carp lengths at age were taken from studies of their growth in a Chinese reservoir at a similar latitude to the Great Lakes (Yan and Shi 1995).

| Age | Silver Carp^1^ | Bighead Carp^1^ | Yellow Perch^2^ | Walleye^2^ | Round Goby^3^ | Rainbow Smelt^4^ |
| --- | --- | --- | --- | --- | --- | --- |
| 1 | 139 ± 5 | 155 ± 10 | 81 ± 5 | 199 ± 10 | 52 ± 5 | 67 ± 5 |
| 2 | 317 ± 5 | 325 ± 10 | 144 ± 5 | 339 ± 10 | 72 ± 5 | 146 ± 5 |
| 3 | 418 ± 5 | 452 ± 10 | 170 ± 5 | 431 ± 10 | 97 ± 5 | 194 ± 5 |
| 4 | 508 ± 5 | 556 ± 15 | 192 ± 10 | 488 ± 15 | 139 ± 5 | 223 ± 5 |
| 5 | 576 ± 10 | 655 ± 15 | 212 ± 10 | 513 ± 15 | 177 ± 5 | 241 ± 5 |
| 6 | 641 ± 10 | 719 ± 15 | 230 ± 10 | 535 ± 15 |  | 252 ± 5 |
| 7 | 699 ± 10 | 762 ± 15 | 245 ± 10 | 564 ± 15 |  |  |
| 8 |  |  | 258 ± 10 | 557 ± 15 |  |  |
| 9 |  |  | 270 ± 10 | 573 ± 15 |  |  |
| 10 |  |  | 280 ± 10 | 589 ± 15 |  |  |
| 11 |  |  | 289 ± 10 | 624 ± 15 |  |  |
| 12 |  |  |  | 624 ± 15 |  |  |

^1^ Yan and Shi (1995)

^2^ Fielder and Thomas (2006)

^3^ Johnson et al. (2005)

^4^ Bailey (1964)

**Temperature**

Daily water temperature was derived from empirical data (Johengen et al. 2000) for Saginaw Bay

For winter (t ≥ 321 and t < 96), T_t_ = 2.0

For other seasons (96 ≤ t < 321)

$T_{t}=-0.0017t^{2}+0.7085t-51.878$ Equation A1

Water temperature in the Saginaw River (Equation A2):

For winter (t ≥ 289 and t < 57), T_t_ = 0.5

For other seasons (57 ≤ t < 289)

$T_{t}= -0.000009t^{3}+0.0032t^{2}-0.1788t+1.9074$ Equation A2

where *T_t_* ( ˚C ) is the temperature on Julian day *t*.

**Biomass pools**

The model included seven biomass pools: phytoplankton, detritus, zooplankton, *Bythotrephes longimanus*, dreissenids, benthos (i.e., amphipods, chironomids, emphemeroptera, hirudinea, isopods, trichoptera, sphaerridae, turbellaria), and forage fish (i.e., cyprinids, Spottail Shiner *Notropis hudsonius*, Emerald Shiner *N. atherinoides*, age 0 Gizzard Shad *Dorosoma cepedianum*, Alewife *Alosa pseudoharengus*, age 0 White Perch *Morone americana*, Trout-perch *Percopsis omiscomaycus* and other prey fish, Kao et al. 2014). Biomass of these groups was carried over from one simulation year to the next. To ensure biomass pools interacted, we used consumption and production equations from EcoPath with EcoSim (EwE). Biomass of prey *j* at day *t* *(B_j,t_*) was updated as:

$B_{j,t}=B_{j,t-1}+P_{j,t}-\sum_{l=1}^{6} {cf}_{j,l,t}-\sum_{k=1}^{m} \left( {Con}_{j,k,t} \right)-{OM}_{j}*B_{j,t-1}$ Equation A3

where *P_j,t_* is the production of biomass pool *j* on day *t*, *cf_j,l,t_* is the consumption by individual fish *l*, *Con_j,k,t_* is the consumption of pool *j* by pool *k,* and *m* represents the total number of consumers for pool *j*, specifically, *m = 5* for phytoplankton and detritus, and *m = 4* for other prey pools. *OM_j_* is “other” mortality as defined in Ecopath of biomass pool *j*, or the decay rate for detritus (Table A1).

Production (*P_phyto,t_*) of phytoplankton was determined as:

$P_{phyto,t}=\left( \frac{P}{B} \right)_{phyto}*B_{phyto}$ Equation A4

where (*P/B)_phyto_* is a production (P) to biomass (B) ratio and *phyto* indicates the phytoplankton biomass pool (Table A3). For phytoplankton, the P/B varied monthly from 0.61-0.91 per day to force seasonality to their growth derived from estimates of production and biomass in Saginaw Bay in 1990 (Fahnenstiel et al. 1995).

Production of detritus (*P_detr,t_*) is derived from unassimilated food through consumption by remaining biomass pools and mortality of other biomass pools, determined as:

$P_{detr,t}=\sum_{k=1}^{5} {(Con}_{detr,k,t}*{ar}_{k})+\sum_{k=1}^{6} {OM}_{k}*B_{k,t-1}$ Equation A5

where *ar_k_* is the unassimilated proportion by consumer pool *k.* , and *detr* indicates the detritus biomass pool.

The production of the remaining biomass pools was determined as:

$P_{k,t}=\frac{\left( \frac{P}{B} \right)_{k}}{\left( \frac{Q}{B} \right)_{k}}*\sum_{j=1}^{6} ({Con}_{j,k,t}*(1-{ar}_{k}))$ Equation A6

where (*Q/B)_k_* is a taxon-specific consumption (Q) to biomass ratio (Table A3). For these groups, consumption (*Con_j,k,t_*) of pool *k* on pool *j* was determined as:

${Con}_{j,k,t}=\frac{a_{j,k}*v_{j,k}*B_{j,t-1}*B_{k,t-1}}{2*v_{j,k}+\left( a_{j,k}*B_{k,t-1} \right)}$ Equation A7

where *a_j,k_* and *v_j,k_* are the respective predator search rate and vulnerability of pool *k* to pool *j*. *a_j,k_* and *v_j,k_* were modified from an EwE model developed for Saginaw Bay (Tables A4 and A5; Kao et al. 2014).

**Table A3**. Initialized values for production to biomass (P/B, per day), consumption to biomass (Q/B, per day), energy density (J g^-1^), unassimilated consumption, other mortality (as defined in EcoPath, per day) sources and initial starting biomass (g m^-2^) for the seven biomass pools. Values for P/B, Q/B, unassimilated consumption, other mortality and biomass estimates were derived from Kao et al. (2014).

| Prey | P/B | Q/B | Energy  Density(J g^-1^) | Unassimilation rate (ar) | Other  Mortality(day^-1^) | Biomass  (g m^-2^) (B) |
| --- | --- | --- | --- | --- | --- | --- |
| Phytoplankton | 0.61-0.91 | - | 1850^1^ | - | 0.265 | 9.64 |
| Detritus | - | - | 1143^1^ | - | 0.005 | 140 |
| Zooplankton | 0.067 | 0.269 | 2300^4^ | 0.420 | 0.043 | 11.74 |
| Bythotrephes | 0.030 | 0.120 | 1674^3^ | 0.400 | 0.018 | 0.01 |
| Benthos | 0.019 | 0.073 | 2427^4^ | 0.500 | 0.011 | 40.16 |
| Dreissenids | 0.006 | 0.042 | 3700^2^ | 0.660 | 0.003 | 5.6 |
| Forage Fish | 0.009 | 0.068 | 5761^1^ | 0.364 | 0.003 | 5.51 |

^1^ Cummins and Wuycheck (1971)

^2^ Johnson et al. (2005)

^3^ Lantry and Stewart (1993)

^4^ Pothoven and Madenjian (2008)

**Table A4**. Search rates (*a*, per day) required for consumption of biomass pools for zooplankton (Zoop), Bythotrephes (Bytho), Benthos, Dreissenids, and Forage fishes (Kao et al. 2014).

| Prey | Zoop | Bytho | Benthos | Dreissenids | | Forage fishes |
| --- | --- | --- | --- | --- | --- | --- |
| Phytoplankton | 9.272 | 0 | 0.273 | 0.442 | 0.500 | |
| Detritus | 3.000 | 0 | 0.082 | 0.019 | 0 | |
| Zooplankton | - | 2.607 | 0 | 0 | 1.778 | |
| Bythotrephes | 0 | - | 0 | 0 | 0 | |
| Benthos | 0 | 0 | - | 0 | 0.051 | |
| Dreissenids | 0 | 0 | 0 | - | 0 | |
| Forage Fishes | 0 | 0 | 0 | 0 | - | |

**Table A5**. Vulnerability (v) values required for consumption of biomass pools (Kao et al. 2014).

| Prey | Zoos | Bythos | Benthos | Dreissenids | Forage Fishes |
| --- | --- | --- | --- | --- | --- |
| Phytoplankton | 1.7 | 0 | 0.01 | 0.4 | 0.01 |
| Detritus | 0.3 | 0 | 0.125 | 0.02 | 0 |
| Zooplankton | 0 | 0.13 | 0 | 0 | 0.05 |
| Bythotrephes | 0 | 0 | 0 | 0 | 0 |
| Benthos | 0 | 0 | 0 | 0 | 0.005 |
| Dreissenids | 0 | 0 | 0 | 0 | 0 |
| Forage Fish | 0 | 0 | 0 | 0 | 0 |

**Individually modeled populations as super individuals (SI)**

Maturation, reproduction, and egg development

Maturation was modeled using species-specific, literature-derived values and was either size-based, age-based or a combination of the two. Yellow Perch and Round Goby were assumed to mature based on length. Yellow Perch mature at 120 mm for males and 170 mm for females (Purchase et al. 2005), while Round Gobies of either sex mature at 60.8 mm (MacInnis and Corkum 2000). Bighead Carp, Silver Carp and Rainbow Smelt were assumed to mature based on age. For Silver Carp, males mature at age 3 while females mature at age 4 (Kolar et al. 2007). Bighead Carp males and females mature at ages 4 and 5, respectively (Kolar et al. 2007). Rainbow Smelt mature at age 2 for males and 3 for females (Bailey 1964). Walleye maturation was derived using published probabilistic maturation reaction norms for males and females (Wang et al. 2009). Males were assumed to mature if they were at least 330 mm or 369 mm at ages 1 or 2 respectively (all age 3 males were assumed mature), while females matured if they were at least 500 mm, 450 mm or 430 mm at ages 2, 3, or 4 respectively (all age 5 females were considered mature).

Temperature and day of the year on which spawning occurred were determined from the literature (Table A6). Spawning temperatures were randomly assigned from a uniform distribution to each SI. Silver Carp, Bighead Carp, Walleye and Rainbow Smelt were assumed to spawn in rivers, while Round Goby and Yellow Perch spawn in Saginaw Bay proper.

Fecundity (*F_i_*_,_ # eggs/female) for SIs was assigned using species-specific fecundity relationships:

$F_{i}=a{*W}_{i}+b$ Equation A8

$F_{i}=a+b{*L}_{i}$ Equation A9

$F_{i}= {10}^{(a{log}_{10} \left( L_{i} \right)-b)}$ Equation A10

where *a* and *b* are species-specific constants and *L_i_* and *W_i_* are the total length (mm) and wet weight (g) of individual *i*, respectively (Table A7). For all species, *F_i_* is then multiplied by *EM_i_*, an egg specific mortality rate (Table A7). Once all females spawned, the total number of new individuals was assessed and then those individuals were divided into 100 new super individuals for each species.

Egg development threshold or time to hatching (*Dv_j,t_*, days) varied amongst species for species *j* and time (*t*, days) (Table A8). Yellow perch, Walleye and Rainbow Smelt egg development was determined based on temperature (T):

$\mathrm{Dv}_{j,t}=\mathrm{Dv}_{j,t-1}+\left( \frac{1}{\left( 145.7+2.56*T_{t}-63.8*\ln\left( T_{t} \right) \right)} \right), \mathrm{Dv}_{j,0}=0$, for Yellow Perch Equation A11

$\mathrm{Dv}_{j,t}=\mathrm{Dv}_{j,t-1}+(\frac{1}{\left( 57.1*\mathrm{Exp}^{\left( \frac{T_{t}}{18.9} \right)}-15.4 \right)})$, $\mathrm{Dv}_{j,0}=0$, for Walleye Equation A12

$\mathrm{Dv}_{j,t}=\mathrm{Dv}_{j,t-1}+\left( 66.293*\mathrm{EXP}^{-0.134*T_{t}} \right), \mathrm{Dv}_{j,0}=0$, for Rainbow Smelt Equation A13

For Yellow Perch and Walleye, eggs hatch when *Dv_j,t_* exceeds a value of 1 (fraction of development, Rose et al. 1999) while rainbow smelt eggs hatch when *Dv_j,t_* exceeds 164.1 accumulated thermal units (° C d, Buckley 1989). Round Goby eggs hatch 18 days after spawning (Marsden et al. 1996). Silver and bighead carp eggs hatch based on accumulated thermal units of 380° C hr and 257.4 °C hr, respectively (Chapman, George 2011) and was calculated as

${Dv}_{j,t}={Dv}_{j,t-1}+\left( T_{t}*24 \right), \mathrm{Dv}_{j,0}=0$ Equation A14

Once hatched, larvae were assigned a length from a normal distribution with a mean and standard deviation (0.5) and fixed number of days till first feeding (Table A8).

**Table A6**. Minimum (Min) and Maximum (Max) spawning temperatures (ºC), spawning day, type of spawner (r = river, s = Saginaw Bay) and migration date (Day of the year) for fish leaving the model environment. Migratory fish moved back into the bay on day 1 of the following year. Spawning temperatures were drawn from a uniform distribution given the minimum and maximum values shown below. For Silver Carp and Bighead Carp, the maximum temperature used in the model differs from the maximum temperature of 30 ºC reported in the literature because temperatures in Saginaw Bay did not exceed 22 ºC in summer.

| Species | Min | Max | Spawn Day | Spawner type | Migration Date |
| --- | --- | --- | --- | --- | --- |
| Silver Carp^1^ | 18 | 21 | 121 | r | - |
| Bighead Carp^1^ | 18 | 21 | 121 | r | - |
| Yellow Perch^2^ | 7 | 11 | 110 | s | - |
| Walleye^2^ | 4 | 13 | 59 | r | 181 |
| Round Goby^3^ | 9 | 21 | 121 | s | - |
| Rainbow Smelt^4^ | 4 | 15 | 60 | r | 151 |

^1^ Kolar et al. (2007)

^2^ Rose et al. (1999)

^3^ Marsden (1996)

^4^ Auer (1982)

**Table A7**. Parameter values for fecundity equations and the egg mortality (%) assigned to each species.

| Species | Fecundity Equation | Fecundity a | Fecundity b | Egg Mortality |
| --- | --- | --- | --- | --- |
| Silver Carp^1^ | A8 | 325.14 | -581908 | 99.0 |
| Bighead Carp^1^ | A8 | 128.47 | -315083 | 99.0 |
| Yellow Perch^2^ | A8 | 183 | -3658 | 91.0 |
| Walleye^2^ | A8 | 70.6 | -7900 | 97.0 |
| Round Goby^3^ | A9 | -331.9 | 8.95 | 40.0 |
| Rainbow Smelt^4^ | A10 | 2.97 | 2.43 | 91.0 |

^1^ DeGrandchamp et al. (2007)

^2^ Rose et al. (1999)

^3^ MacInnis and Corkum (2000)

^4^ Lantry and Stewart (1993)

**Table A8**. Development threshold or time for eggs to hatch, mean and standard deviation (SD) of larval length (mm) at first feeding, and time to first feeding. Development to hatch and time to first feeding varied with species and were accumulated over time. Summations were accumulated thermal units (°C hr) for Silver and Bighead Carp, and Rainbow Smelt (°C d). Summations were numerical and had to exceed a value of 1 for Walleye and Yellow Perch, but were summed days for round goby.

| Species | Development to hatch | Mean Length and SD (mm) | Time to First Feeding |
| --- | --- | --- | --- |
| Silver Carp^1^ | 380 (°C hr) | 10 (0.5) | 1680 (°C hr) |
| Bighead Carp^1^ | 257.4 (°C hr) | 10 (0.5) | 1333.2 (°C hr) |
| Yellow Perch^2^ | 1 | 7.5 (0.5) | 4 d |
| Walleye^2^ | 1 | 9.5 (0.5) | 2 d |
| Round Goby^3^ | 18 d | 6.2 (0.5) | 21 d |
| Rainbow Smelt^4^ | 164.1 (°C d) | 6.4 (0.5) | 204.1 (°C d) |

^1^ Chapman and George (2011)

^2^ Rose et al. (1999)

^3^ MacInnis and Corkum (2000)

^4^ Auer (1982)

**Bioenergetic growth**

Daily growth was determined using the Wisconsin-type Bioenergetic modes (Hanson et al. 1997, Equations A15 - A33) as:

$W_{i}= C_{i}-R_{i}-F_{i}-U_{i}-S_{i}$ Equation A15

where *W_i_* is wet weight (g), *C_i_* (g g^-1^ d^-1^) is total consumption of all prey types by individual *i,* *R_i_* is respiration (g g^-1^ d^-1^), *F_i_* is egestion (g g^-1^ d^-1^), *U_i_* (g g^-1^ d^-1^) is excretion, and *S_i_* (g g^-1^ d^-1^) is specific dynamic action.

To determine consumption, it was necessary to calculate *C_max_* (maximum consumption in g g^-1^ d^-1^) to cap the consumption estimated from the foraging model:

${Cmax}_{i}=CA*{W_{i}}^{CB}$ Equation A16

where CA and CB are the weight-dependent coefficients for each species (Table A9) with energy densities in Table A10.

Consumption (*C_i.j_*) by individual fish was based on a multispecies type II feeding functional response:

$C_{i,j}=\frac{{Cmax}_{i} {Vul}_{i,j} B_{j}a_{i,j}/K_{i,j}}{1+\sum_{j}^{6} {Vul}_{i,j} B_{j} a_{i,j}/K_{i,j}}$ Equation A17

where *Cmax_i_* is the maximum consumption from the bioenergetics model of individual *i*, *Vul_i,j_* is the vulnerability of prey species *j* to individual *i*, *B_j_* is the biomass of prey *j* available to individual *i* which was updated after each simulation day, *a_i,j_* is a parameter representing the search and encounter of prey type *j* by individual *i*, and *K_i,j_* is the half saturation constant of fish species *i* feeding on prey *j*. *a_i,j_/K_i,j_* was fit during baseline model runs such that diets reflected general diets of Walleye, Yellow Perch, Round Goby and Rainbow Smelt with ontogeny (Table A11). The value of a_i,j_/K_i,j_ was then allowed to randomly vary for each individual each day by ± 25%. Biomass pools hold reserve values that cannot be consumed by individuals or other biomass pools to reflect the theory of foraging arena (Ahrens et al. 2011). Vulnerabilities of biomass pools are based on literature information and may vary with size (e.g., Yellow Perch greater than 30 mm may consume benthos while those less than 30 mm cannot) or may remain static (e.g., phytoplankton are never vulnerable to Yellow Perch predation). Vulnerability of pools to individuals was either 0 or 1, but whether a pool was vulnerable to a specific species varied with fish length (Table A12). The exception to this rule was for Walleye and Round Goby preying on zooplankton, which were given maximum fish lengths after which zooplankton were no longer included in their diets (Table A12).

Vulnerability of SIs that are consumed by other SIs is more complex than vulnerability of biomass pools to SI fishes. Values for vulnerability of individual fishes ranged from 0-1 and were assigned either based on the ratio of prey to predator length or prey length only. For silver and bighead carp, prey length (*pl_j_*) was used to determine vulnerability of individual larval fishes to predation by carps. Vulnerability of fish *j* decreased as its length increased up to 12 mm, when it was no longer vulnerable to predation by carps:

$V_{i,j}=-0.278*{pl}_{j}^{2}+0.333*{pl}_{j}$ Equation A18

Vulnerability of fish *j* to predation by Yellow Perch, Walleye, Round Goby, and Rainbow Smelt (*i*) was based on the ratio of prey length to predator length (*R_i,j_*). For Yellow Perch, Rainbow Smelt and Round Goby, vulnerability was calculated as:

$V_{i,j}=-400*R_{i,j}^{2}+40*R_{i,j}$ Equation A19

For Walleye, this relationship was:

$V_{i,j}=-64*R_{i,j}^{2}+22.4*R_{i,j}-0.96$ Equation A20

Fish were no longer vulnerable to predation when the ratio of prey to predator length was greater than 0.1 for Yellow Perch, Rainbow Smelt and Round Goby and 0.3 for Walleye. Further, Walleye were unable to eat prey less 0.05 of their length.

Equations used to model respiration (*R_i_*) were species specific (Table A9):

$R_{i}=RA*{W_{i}}^{RB}*f(T)*ACT*Oxy$ Equation A21

$R_{i}=RA*{W_{i}}^{RB}*{EXP}^{\left( RQ*T \right)}*Oxy$ Equation A22

where RA and RB are the intercept and the slope of the allometric mass function, *f(T)* is a temperature dependent function, ACT is an activity multiplier, RQ approximates the Q_10_ rate, and Oxy is the oxicalorific coefficient used to convert units from oxygen consumption to joules. For equation A17, *f(T)* was defined as:

$f(T)=v^{x}*{Exp}^{(x*\left( 1-v \right))}$ Equation A23

$v=(RTM-T_{d})/(RTM-RTO)$ Equation A24

$z=ln(RQ)*(RTM-RTO)$ Equation A25

$y=\ln\left( RQ \right)*(RTM-RTO+2)$ Equation A26

$x=\frac{(z^{2}*{(1+\left( 1+\frac{40}{y} \right)^{0.5})}^{2}}{400}$ Equation A27

where RTO is the coefficient for swimming speed dependence of metabolism, RTM is the maximum lethal water temperature (°C).

Likewise, equations for egestion (*F_i_*) and excretion (*U_i_*) are species-specific and follow two different forms (Table A9).

$F_{i}=FA*C_{i}$ Equation A28

$U_{i}=UA(C_{i}-F_{i})$ Equation A29

where FA and UA are constant proportions of egestion and excretion or

$F_{i}=FA*T^{FB}*{Exp}^{\left( FG*{Pval}_{i} \right)}*C_{i}$ Equation A30

$U_{i}=UA*T^{UB}*{Exp}^{\left( UG*{Pval}_{i} \right)}(C_{i}-F_{i})$ Equation A31

where FA and UA are the intercepts of the proportion of energy egested or excreted, FB and UB is the coefficient of water temperature dependence of egestion and excretion, FG and UG are the coefficients for the feeding level dependence of egestion and excretion, and *Pval_i_* is the proportion of maximum consumption by individual *i*. Specific dynamic action (*S_i_*), is then determined as:

$S_{i}=SDA*\left( C_{i}-F_{i} \right)$ Equation A32

where SDA is the constant.

Finally, individuals could only lose weight not length. If a fish’s weight increased, a new length (mm) was calculated as

$L_{i,t}={(\frac{W_{i,t}}{lwa})}^{\frac{1}{lwb}}$ Equation A33

where lwa and lwb are length-weight parameters (Table A9).

Energy density of SI’s is stage dependent. We define stage 1 as age-0 fishes < 20mm; stage-2 as age-0 fishes ≥ 20mm; stage 3 fishes age-1+ are immature, while stage 4 fishes age 1+ are mature (Table A10).

**Table A9**. Species-specific bioenergetics equations and parameters values and length-weight parameters. Parameters values for different life history stages are shown where stages are defined as: age-0 fishes less than 20mm stage 1, age-0 fishes greater than 20mm stage 2, stage 3 fishes are immature while stage 4 fishes are mature. For maximum consumption, respiration, egestion and excretion the equations are shown for each species. Oxycalorific conversion factor used is 13,560 J g^-1^ O_2_  (Stewart et al. 1983).

| Species | Silver Carp^1,2^ | Bighead Carp^1,3^ | Yellow Perch^4,5^ | | | Walleye^5,6,7,8^ | | Round Goby^9,10^ | Rainbow Smelt^4,11^ |
| --- | --- | --- | --- | --- | --- | --- | --- | --- | --- |
| Stage | All | All | stage 1 | stage 2 | stage 3,4 | stage 1 | stage 2,3,4 | All | All |
| *CA* | 1.54 | 1.54 | 0.51 | 0.25 | 0.25 | 0.45 | 0.25 | 0.192 | 0.18 |
| *CB* | -0.287 | -0.287 | -0.42 | -0.27 | -0.27 | -0.27 | -0.27 | -0.256 | -0.275 |
| Respiration | A22 | A22 | A21 | A21 | A21 | A21 | A21 | A22 | A22 |
| *RA* | 0.0028 | 0.0053 | 0.0065 | 0.0108 | 0.0108 | 0.0138 | 0.0108 | 0.00094 | 0.0027 |
| *RB* | -0.239 | -0.299 | -0.2 | -0.2 | -0.2 | -0.22 | -0.2 | -0.157 | -0.216 |
| *RQ* | 0.076 | 0.048 | 2.1 | 2.1 | 2.1 | 2.1 | 2.1 | 0.061 | 0.036 |
| *RTO* |  |  | 32 | 32 | 28 | 27 | 27 |  |  |
| *RTM* |  |  | 35 | 35 | 33 | 32 | 32 |  |  |
| *ACT* | 1.0 | 1.0 | 4.4 | 1.0 | 1.0 | 3.3 | 1.0 | 1.0 | 1.0 |
| *SDA* | 0.1 | 0.1 | 0.15 | 0.172 | 0.172 | 0.1 | 0.172 | 0.175 | 0.175 |
| Egestion | A30 | A30 | A28 | A30 | A30 | A28 | A30 | A28 | A28 |
| Excretion | A31 | A31 | A29 | A31 | A31 | A29 | A31 | A29 | A29 |
| *FA* | 0.212 | 0.212 | 0.15 | 0.158 | 0.158 | 0.25 | 0.158 | 0.15 | 0.16 |
| *FB* | -0.222 | -0.222 |  | -0.222 | -0.222 |  | -0.222 |  |  |
| *FG* | 0.631 | 0.631 |  | 0.631 | 0.631 |  | 0.631 |  |  |
| *UA* | 0.031 | 0.031 | 0.15 | 0.0253 | 0.0253 | 0.05 | 0.0253 | 0.1 | 0.1 |
| *UB* | 0.58 | 0.58 |  | 0.58 | 0.58 |  | 0.58 |  |  |
| *UG* | -0.299 | -0.299 |  | -0.299 | -0.299 |  | -0.299 |  |  |
| *lwa* (×10^-5^) | 4.0 | 4.0 | 2.36 | 2.36 | 2.36 | 0.422 | 0.422 | 0.6 | 0.122 |
| *lwb* | 2.9003 | 2.8881 | 2.888 | 2.888 | 2.888 | 3.123 | 3.123 | 3.185 | 3.315 |

^1^ Cooke and Hill (2010)

^2^ Garvey et al. (2007)

^3^ Schrank and Guy (2002)

^4^ Hanson et al. (1997)

^5^ Rose et al. (1999)

^6^ Lantry et al. (2008)

^7^ Madon and Culver (1993)

^8^ Minton and McLean (1982)

^9^ Lee and Johnson (2005)

^10^ MacInnis and Corkum (2000)

^11^ Isaac (2010)

**Table A10**. Energy density (J g^-1^) of fish life stages for SIs. Age-0 fishes less than 20mm are stage 1, greater than 20mm stage 2, stage 3 fishes are immature while stage 4 fishes are mature.

| Species | Stage 1 | Stage 2 | Stage 3 | Stage 4 |
| --- | --- | --- | --- | --- |
| Silver Carp^1^ | 5,442 | 5,442 | 5,442 | 5,442 |
| Bighead Carp^1^ | 5,442 | 5,442 | 5,442 | 5,442 |
| Yellow Perch^2^ | 2,512 | 4,186 | 4,186 | 4,186 |
| Walleye^3^ | 3,349 | 5,587 | 5,587 | 5,587 |
| Round Goby^4^ | 4,600 | 4,600 | 4,600 | 4,600 |
| Rainbow Smelt^2^ | 3,416 | 4,102 | 4,814 | 4,814 |

^1^ Cooke and Hill (2010)

^2^ Hanson et al. (1997)

^3^ Madon and Culver (1993)

^4^ Lee and Johnson (2005)

**Table A11.** Parameter values of the ratio of search (a) to half saturation rate (K) (where *a* is the search and encounter parameter and *K* is the half-saturation parameter) for biomass pools available to predator super individuals (SIs). The a/K values for Yellow Perch, Walleye, Round Goby and Rainbow Smelt were fitted during baseline calibration without bigheaded carps included in the model, while those of bigheaded carp were fitted in runs with only carp in the system and no other fish.

| Species | Biomass Pools | Stage 1 | Stage 2 | Stage 3 | Stage 4 |
| --- | --- | --- | --- | --- | --- |
| Silver Carp | Phytoplankton | 0.01 | 0.025 | 0.024 | 0.015 |
| Silver Carp | Detritus | 0.001 | 0.002 | 0.0025 | 0.001 |
| Silver Carp | Zooplankton | 0.08 | 0.001 | 0.0001 | 0.0001 |
| Silver Carp | Bythotrephes | 0.0001 | 0.0001 | 0.0001 | 0.0001 |
| Silver Carp | Benthos | 1 | 1 | 1 | 1 |
| Silver Carp | Dreissenids | 1 | 1 | 1 | 1 |
| Silver Carp | Forage Fish | 1 | 1 | 1 | 1 |
| Silver Carp | Individual Fish | 1 | 1 | 1 | 1 |
| Bighead Carp | Phytoplankton | 0.01 | 0.001 | 0.0001 | 0.0001 |
| Bighead Carp | Detritus | 0.001 | 0.002 | 0.0025 | 0.001 |
| Bighead Carp | Zooplankton | 0.1 | 0.027 | 0.013 | 0.007 |
| Bighead Carp | Bythotrephes | 0.0001 | 0.0001 | 0.0001 | 0.0001 |
| Bighead Carp | Benthos | 1 | 1 | 1 | 1 |
| Bighead Carp | Dreissenids | 1 | 1 | 1 | 1 |
| Bighead Carp | Forage Fish | 1 | 1 | 1 | 1 |
| Bighead Carp | Individual Fish | 1 | 1 | 1 | 1 |
| Yellow Perch | Phytoplankton | 1 | 1 | 1 | 1 |
| Yellow Perch | Detritus | 1 | 1 | 1 | 1 |
| Yellow Perch | Zooplankton | 0.06 | 0.06 | 0.0025 | 0.001 |
| Yellow Perch | Bythotrephes | 0.0001 | 0.001 | 0.01 | 0.01 |
| Yellow Perch | Benthos | 0.0001 | 0.013 | 0.05 | 0.01 |
| Yellow Perch | Dreissenids | 1 | 1 | 1 | 1 |
| Yellow Perch | Forage Fish | 0.000001 | 0.00001 | 0.03 | 0.075 |
| Yellow Perch | Individual Fish | 0.000001 | 0.00001 | 0.03 | 0.05 |
| Walleye | Phytoplankton | 1 | 1 | 1 | 1 |
| Walleye | Detritus | 1 | 1 | 1 | 1 |
| Walleye | Zooplankton | 0.5 | 0.1 | 0.00001 | 0 |
| Walleye | Bythotrephes | 0.005 | 0.002 | 0.0005 | 0 |
| Walleye | Benthos | 0.15 | 0.4 | 0.02 | 0.00075 |
| Walleye | Dreissenids | 1 | 1 | 1 | 1 |
| Walleye | Forage Fish | 0.1 | 0.6 | 0.2 | 0.125 |
| Walleye | Individual Fish | 0.1 | 0.6 | 0.35 | 0.55 |
| Round Goby | Phytoplankton | 1 | 1 | 1 | 1 |
| Round Goby | Detritus | 1 | 1 | 1 | 1 |
| Round Goby | Zooplankton | 0.7 | 0.002 | 0.00002 | 0.00002 |
| Round Goby | Bythotrephes | 0.005 | 0.0001 | 0.00002 | 0.00002 |
| Round Goby | Benthos | 0.04 | 0.04 | 0.02 | 0.005 |
| Round Goby | Dreissenids | 0.05 | 0.075 | 0.05 | 0.125 |
| Round Goby | Forage Fish | 0.00001 | 0.00001 | 0.00001 | 0.00001 |
| Round Goby | Individual Fish | 0.0000001 | 0.00001 | 0.00001 | 0.00001 |
| Rainbow Smelt | Phytoplankton | 1 | 1 | 1 | 1 |
| Rainbow Smelt | Detritus | 1 | 1 | 1 | 1 |
| Rainbow Smelt | Zooplankton | 0.1 | 0.125 | 0.025 | 0.04 |
| Rainbow Smelt | Bythotrephes | 0.0001 | 0.005 | 1 | 1 |
| Rainbow Smelt | Benthos | 0.000001 | 0.000001 | 0.000001 | 0.000001 |
| Rainbow Smelt | Dreissenids | 1 | 1 | 1 | 1 |
| Rainbow Smelt | Forage Fish | 0.00000001 | 0.0001 | 0.0001 | 0.0001 |
| Rainbow Smelt | Individual Fish | 0.00000001 | 0.0001 | 0.0001 | 0.0001 |

**Table A12**. Length ranges (mm) of SI fish where the prey pools became vulnerable to predation by the SI fish. “-” represents invulnerability of biomass pools to individual fishes, while “>0” represents that fish can always consume the prey pools.

| Species | Phyto | Detritus | Zoos | Bythos | Benthos | Dreissenids | Forage fish |
| --- | --- | --- | --- | --- | --- | --- | --- |
| Silver Carp | >0 | >0 | >0 | >17 | - | - | - |
| Bighead Carp | >0 | >0 | >0 | >17 | - | - | - |
| Yellow Perch | - | - | >0 | >30 | >30 | - | >200 |
| Walleye | - | - | <60 | >15 | >15 | - | >30 |
| Round Goby | - | - | <45 | >20 | >20 | >60 | - |
| Rainbow Smelt | - | - | 0 | >30 | >45 | - | >141 |

**Mortality**

Super individual fish (SIs) can experience three types of mortality: starvation, predation by SIs, and other mortality (or background mortality). In general, the starvation threshold at which fish die due to starvation ranges from 58-87% (Letcher et al. 1996). Because we used super individuals, we chose not to remove all individuals represented by a SI at once. Instead, starvation mortality occurred when an individual’s weight dropped 50% below its expected weight given its length. If this occurred, 30% of the individuals represented by the SI were removed. If a SI’s weight dropped below 10% of its expected weight, all remaining individuals represented by the SI were removed. Predation mortality occurs when a SI is preyed upon by another SI. In this case, the number of individuals removed from an SI is dependent on the grams consumed by the predatory SI. Finally, to mimic other mortality, individuals were removed from SIs as:

${N_{t+1}}_{i}={N_{t}}_{i}(1- Z_{i})$ Equation A34

where *N_t+1i_* is the number of individuals represented by an SI at time *t+1*, and *Z_i_* is background daily instantaneous mortality. In fishes ≥0.1 g, *Z_i_* is weight-specific (Lorenzen 1996):

$Z_{i}=\frac{3.13*W_{i}^{-0.309}}{365}*Mod$ +Addmort Equation A35

where *W_i_* is mass (g) of fish *i*, *Mod* is a species- and size-specific modifier on mortality (Table A13), and *Addmort* is an additional mortality*.* Mature bigheaded carp, Walleye and Yellow Perch experienced additional mortality (Addmort) on top of the mortality generated by Lorenzen’s rates (1996) in order to capture mortality in older fishes due to the fishing and spawning mortality. For fish <0.1g, a base *Z* rate of 0.85×Mod was assigned (Houde 2002; Table A13).

**Table A13**. Calibrated values for modifiers of daily instantaneous mortality Z for each species for fish <0.1g, ≥0.1g, and for additional mortality (caused by spawning or fishing) in Walleye, Yellow Perch, and bigheaded carp. For bigheaded carp, the multipliers resulted in low, intermediate, and high age-0 survival rates. The small additive mortality differed by age of Yellow Perch and Walleye, decreasing for age-3 and older Yellow Perch, and increasing for age-7 and older Walleye.

| Species | <0.1g | ≥0.1g | Additional Mort. |
| --- | --- | --- | --- |
| Silver carp | 2.5, 1.4 , 0.1 | 1.15 | 7.3E-4 |
| Bighead carp | 2.5, 1.4 , 0.1 | 1.15 | 7.3E-4 |
| Yellow perch | 1.2 | 0.8 | 2.9E-4,7.8E-5 |
| Walleye | 0.61 | 0.65 | 9.4E-4, 1.1E-3 |
| Round goby | 0.05 | 1.1 |  |
| Rainbow smelt | 0.11 | 0.01 |  |

**Movement**

We simulated two types of movement for SIs in the model. One movement was for spawning migrations, and the other was for migrations that removed SIs from the model domain (i.e., leave Saginaw Bay to Lake Huron. The type of spawning migration depended on the spawning classification of individual species. Spawning migrations commenced when two conditions were met: the initial spawning date was exceeded and the water temperature that initiated spawning was within 2°C of the SI’s assigned spawning temperature, which was randomly assigned from a normal distribution (Table A6). Once these conditions were met, fish were moved to their assigned spawning grounds. We simulated spawning by species that home to a natal river to spawn (Walleye and Rainbow Smelt), by species that spawn in rivers but do not home (Silver and Bighead Carp), and by species that spawn in Saginaw Bay (Yellow Perch and Round Goby). Walleye and Rainbow Smelt were assigned one of two spawning rivers. After initialization, the assigned river was dependent on their mother’s assigned river. For bigheaded carps, there was no information on the likelihood of migrating from a lake to spawn in rivers. Therefore, we assumed there was a 50% probability of a mature fish finding a river where it could spawn. Once Walleye and Rainbow Smelt spawned, they moved into Saginaw Bay. Yellow Perch and Round Goby spawned within the bay itself once the assigned spawning temperature was reached.

In the second type of movement, migratory fish left the model domain. Annually, fishes were assigned either migrant or nonmigrant status. Nonmigrants did not leave the model domain. Migrants, however, spent the rest of the year growing and surviving in boundary cells (Lake Huron) (Table A14). Migratory fish moved back into the bay on day 1 of the following year. Walleye and Rainbow Smelt were defined as migrants. Walleye > age 3 had a fifty percent probability of being migratory in any given year. All age-1+ Rainbow Smelt migrated out of the bay each year. Walleye migrated out on Julian day 181 and Rainbow Smelt on Julian day 120 to Lake Huron.

**Table A14**. Values for annual increases in weight gained (g) and annual survival rates (%) for Walleye (Fielder and Thomas 2006) and Rainbow Smelt (Bailey 1964) during their migration period outside the model domain.

| Age | Walleye Weight Gained (g) | Walleye Survival (%) | Rainbow Smelt Weight Gained (g) | Rainbow Smelt Survival (%) |
| --- | --- | --- | --- | --- |
| 1 | NA | NA | 17 | 50 |
| 2 | 599 | 52 | 28 | 54 |
| 3 | 555 | 58 | 28 | 84 |
| 4 | 284 | 64 | 22 | 41 |
| 5 | 291 | 68 | 16 | 24 |
| 6 | 408 | 69 | 10 | 12 |
| 7 | 456 | 70 | NA | NA |
| 8 | 277 | 62 | NA | NA |
| 9 | 294 | 79 | NA | NA |
| 10+ | 311 | 55 | NA | NA |

**Supplement B: Model calibration Results**

Observed data on prey biomass from 2008-2011 for model calibration were based on field surveys of Saginaw Bay (Stow and Hook 2013). Size-at-age and abundance estimates for Yellow Perch and Walleye were derived from Saginaw Bay trawl and gill net data from 1998 to 2004 (Fielder and Thomas 2006). Round Goby data were derived using P/B values from Kao et al. (2014) with lengths at age from Johnson et al. (2005). Rainbow Smelt length at age and maturity values were derived from Bailey (1964).

Model simulated lengths at age matched observed values well for all fish species and ages observed except for Yellow Perch, which were slightly larger than observed at older ages (Figure B1). Simulated abundances were similar to observed values for Yellow Perch and Walleye, but approximately 30% higher than observed for Rainbow Smelt and ~125% greater for Round Goby (Figure B2). For bigheaded carp scenarios, simulated growth of Bighead and Silver Carp was similar to rates reported by Yan and Shi (1995) (Figure B3).

**Figure B1**. Model calibration on simulated length (mm TL) at age (Mod) for Yellow Perch, Walleye, Round Goby and Rainbow Smelt in Saginaw Bay, Lake Huron. Obs indicated observation values.

**Figure B2**. Observed and simulated mean abundances (numbers ± SE) of fish species for the first 5 years (B) on day 300. Model simulations are in black and observations are in gray.


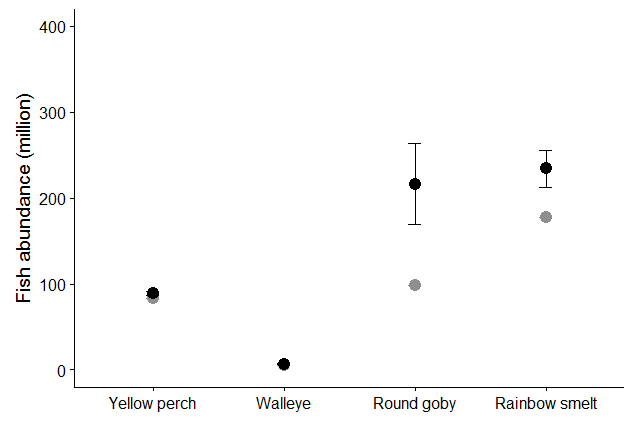


**Figure B3**. Model calibration. Model predicted (dots) and observed lengths (lines) at age for Silver Carp (SC, black) and Bighead Carp (BC, gray). Lines represent lengths at age calculated from a meta-analysis of bigheaded carp vital rates by Tsehaye et al. (2013), and dashed lines represent lengths at age reported by Yan and Shi (1995).

**Simulated prey pool biomass**

**Figure B4**. Biomass (grams wet weight m^-2^) of phytoplankton, zooplankton, detritus, Bythotrephes, forage fish, benthos and dreissenid mussels for the baseline scenario without bigheaded carp (BaseL), the **high** age-0 survival Bighead Carp scenario (BH), and the **high** age-0 survival Silver Carp (SH) scenarios. Simulated biomass values for prey pools exhibited expected seasonal changes.

**Figure B5.**  Biomass (grams wet weight m^-2^) of phytoplankton, zooplankton, detritus, Bythotrephes, forage fish, benthos and dreissenid mussels under for the baseline scenario without bigheaded carp (BaseL), the **intermediate** age-0 survival Bighead Carp scenario (BM), and the **intermediate** age-0 survival Silver Carp (SM) scenarios. Simulated biomass values for prey pools exhibited expected seasonal changes.

**Figure B6** Biomass (grams wet weight m^-2^) of phytoplankton, zooplankton, detritus, Bythotrephes, forage fish, benthos and dreissenid mussels under for the baseline scenario without bigheaded carp (BaseL), the **low** age-0 survival Bighead Carp scenario (BL), and the **low** age-0 survival Silver Carp (SL) scenarios. Simulated biomass values for prey pools exhibited expected seasonal changes

**References**

Ahrens RNM, Wlters CJ, Christensen V (2011) Foraging arena theory. Fish and Fisheries DOI: 10.111/j.1467-2979.2011.00432.x

Auer NA (1982) Identification of larval fishes of the Great Lakes basin with emphasis on the Lake Michigan drainage. Great Lakes Fishery Commission, Ann Arbor, MI 48105 Special Pub 82-3:744 pp

Bailey MM (1964) Age, Growth, Maturity, and Sex Composition of the American Smelt, Osmerus mordax (Mitchill), of Western Lake Superior. Trans Am Fish Soc 93:382-395 doi:doi:10.1577/1548-8659(1964)93[382:AGMASC]2.0.CO;2

Buckley JL (1989) Species profiles: life histories and environmental requirements of coastal fishes and invertebrates (North Atlantic)—rainbow smelt. US Fish and Wildlife Service, Report 82(11.106), US Army Corps of Engineers, TR EL-82-4, 11pp.

Chapman DC, George AG (2011) Developmental rate and behavior of early life stages of bighead carp and silver carp. US Geological Survey Scientific Investigations Report 2011-5076, 11p.

Christensen V, Walters CJ (2004) Ecopath with Ecosim: methods, capabilities and limitations. Ecol Model 172:109-139

Cooke SL, Hill WR (2010) Can filter-feeding Asian carp invade the Laurentian Great Lakes? A bioenergetic modelling exercise. Freswat Biol 55:2138-2152 doi:10.1111/j.1365-2427.2010.02474.x

Cumminns KW, Wuycheck JC (1971) Caloric Equivalents for Investigations in Ecological Energetics. SIL Communications, 1953-1996 18:1-158 doi:10.1080/05384680.1971.11903918

DeGrandchamp KL, Garvey JE, Csoboth LA (2007) Linking adult reproduction and larval density of invasive carp in a large river. Trans Am Fish Soc 136:1327-1334 doi:Doi 10.1577/T06-233.1

Fahnenstiel GL, Bridgeman TB, Lang GA, McCormick MJ, Nalepa TF (1995) Phytoplankton productivity in Saginaw Bay, Lake Huron: Effects of zebra mussel (Dreissena polymorpha) colonization. J Great Lakes Res 21:465-475

Fielder DG, Thomas MV (2006) Fish population dynamics of Saginaw Bay, Lake Huron 1998-2004. Michigan Department of Natural Resources, Fisheries Research Report 2083, Ann Arbor MI. http://www.michigandnr.com/Publications/PDFS/IFR/ifrlibra/Research/reports/2083rr.pdf.

Garvey JE, DeGrandchamp KL, Williamson CJ (2007) Life history attributes of Asian carps in the Upper Mississippi River system. Aquatic Nuisance Species Research Program, ERDC/TN ANSRP-07-1.

Hanson PC, Johnson TB, Schindler DE, Kitchell JF (1997) Fish bioenergetics 3.0. University of Wisconsin System Sea Grant Institute, Madison

Houde ED (2002) Chapter 3. Mortality. In: Fuiman LA, Werner RG (eds) Fishery science: The unique contribution of early life stages. Blackwell Scientific Publishing, Oxford,

Issac EJ (2010) An evaluation of the importance of *Mysis relicta* to the Lake Superior Fish Community. University of Minnesota

Johengen T, Nalepa T, Lang GA, Fanslow D, Vanderploeg H, Agy M (2000) Physical and chemical variables of Saginaw Bay, Lake Huron in 1994-1996. NOAA Technical Memorandum, GLERL-115, Ann Arbor, MI. https://www.glerl.noaa.gov/pubs/tech_reports/glerl-115/tm-115.pdf.

Johnson TB, Bunnell DB, Knight CT (2005) A potential new energy pathway in central Lake Erie: the round goby connection. J Great Lakes Res 31:238-251

Kao Y-C, Adlerstein S, Rutherford E (2014) The relative impacts of nutrient loads and invasive species on a Great Lakes food web: An Ecopath with Ecosim analysis. J Great Lakes Res 40, Supplement 1:35-52 doi:http://dx.doi.org/10.1016/j.jglr.2014.01.010

Kolar CS, Chapman DC, Courtenay Jr. WR, Housel CM, Williams JD, Jennings DP (2007) Bigheaded carps: a biological synopsis and environmental risk assessment American Fisheries Society Special Publication 33, Bethesda, Maryland

Lantry BF, Rudstam LG, Forney JL, VanDeValk AJ, Mills EL, Stewart DJ, Adams JV (2008) Comparisons between Consumption Estimates from Bioenergetics Simulations and Field Measurements for Walleyes from Oneida Lake, New York. Trans Am Fish Soc 137:1406-1421 doi:doi:10.1577/T07-051.1

Lantry BF, Stewart DJ (1993) Ecological energetics of rainbow smelt in the Laurentian Great Lakes - an interlake comparison. Trans Am Fish Soc 122:951-976

Lee VA, Johnson TB (2005) Development of a bioenergetics model for the round goby (Neogobius melanostomus). J Great Lakes Res 31:125-134

Letcher BH, Rice JA, Crowder LB, Rose KA (1996) Variability in survival of larval fish: disentangling components with a generalized individual-based model. Can J Fish Aquat Sci 53:787-801 doi:10.1139/f95-241

Lorenzen K (1996) The relationship between body weight and natural mortality in juvenile and adult fish: a comparison of natural ecosystems and aquaculture. J Fish Biol 49:627-647

MacInnis AJ, Corkum LD (2000) Fecundity and Reproductive Season of the Round Goby Neogobius melanostomus in the Upper Detroit River. Trans Am Fish Soc 129:136-144 doi:10.1577/1548-8659(2000)129<0136:FARSOT>2.0.CO;2

Madon SP, Culver DA (1993) Bioenergetics Model for Larval and Juvenile Walleyes: An in Situ Approach with Experimental Ponds. Trans Am Fish Soc 122:797-813 doi:doi:10.1577/1548-8659(1993)122<0797:BMFLAJ>2.3.CO;2

Marsden JE, Charlebois PM, Wolfe AM, Jude DJ, Rudnicka S (1996) The Round Goby (Neogobius melanostomus): A review of European and North American literature. https://www.ideals.illinois.edu/bitstream/handle/2142/10174/inhscaev01996i00010_opt.pdf?s. Aquatic Ecology Technical Report 96/10, Illinois Natural History Survey.

Minton JW, McLean RB (1982) Measurements of Growth and Consumption of Sauger (Stizostedion canadense): Implication for Fish Energetics Studies. Can J Fish Aquat Sci 39:1396-1403 doi:10.1139/f82-188

Pauly D, Christensen V, Walters C (2000) Ecopath, Ecosim, and Ecospace as tools for evaluating ecosystem impact of fisheries. ICES J Mar Sci 57:697-706

Pothoven SA, Madenjian CP (2008) Changes in consumption by alewives and lake whitefish after dreissenid mussel invasions in Lakes Michigan and Huron. N Am J Fish Manage 28:308-320 doi:10.1577/m07-022.1

Purchase CF, Collins NC, Morgan GE, Shuter BJ (2005) Sex-specific covariation among life-history traits of yellow perch (*Perca flavescens*). Evol Ecol Res 7:549-566

Rose KA, Rutherford ES, McDermot DS, Forney JL, Mills EL (1999) Individual-based model of yellow perch and walleye populations in Oneida Lake. Ecol Monogr 69:127-154

Scheffer M, Baveco JM, Deangelis DL, Rose KA, Vannes EH (1995) Super-Individuals a Simple Solution for Modeling Large Populations on an Individual Basis. Ecol Model 80:161-170 doi:Doi 10.1016/0304-3800(94)00055-M

Schrank SJ, Guy CS (2002) Age, Growth, and Gonadal Characteristics of Adult Bighead Carp, Hypophthalmichthys nobilis, in the Lower Missouri River. Environ Biol Fish 64:443-450 doi:10.1023/a:1016144529734

Stewart DJ, Weininger D, Rottiers DV, Edsall TA (1983) An Energetics Model for Lake Trout, Salvelinus namaycush: Application to the Lake Michigan Population. Can J Fish Aquat Sci 40:681-698 doi:10.1139/f83-091

Stow CA, Hook TO (2013) SAGINAW BAY Multiple Stressors Summary Report, NOAA Technical Memorandum, GLERL-160. Ann Arbor, MI (https://www.glerl.noaa.gov/pubs/tech_reports/glerl-160/tm-160.pdf)

Tsehaye I, Catalano M, Sass G, Glover D, Roth B (2013) Prospects for Fishery-Induced Collapse of Invasive Asian Carp in the Illinois River. Fisheries 38:445-454 doi:10.1080/03632415.2013.836501

Wang H-Y, Cook HA, Einhouse DW, Fielder DG, Kayle KA, Rudstam LG, Höök TO (2009) Maturation Schedules of Walleye Populations in the Great Lakes Region: Comparison of Maturation Indices and Evaluation of Sampling-Induced Biases. N Am J Fish Manage 29:1540-1554 doi:10.1577/M08-156.1

Yan Z, Shi W (1995) Growth and growth models of silver carp and bighead carp in Dahuofang Reservoir. Journal of Fisheries of China 19:28-34 (in Chinese)

Zhang H et al. (2016) Forecasting the Impacts of Silver and Bighead Carp on the Lake Erie Food Web. Trans Am Fish Soc 145:136-162 doi:10.1080/00028487.2015.1069211
